# Textile suit for anywhere full-body motion capture

**DOI:** 10.1126/sciadv.aea2646

**Published:** 2026-03-04

**Authors:** Huanbo Sun, Yao Feng, Pei-Chun Kao, Michael J. Black, Rebecca Kramer-Bottiglio

**Affiliations:** ^1^ Yale University, New Haven, CT, USA.; ^2^ Haptic Sensing Lab, Peking University, Beijing, CN.; ^3^ Max Planck Institute for Intelligent Systems, Tübingen, Germany.; ^4^ ETH Zürich, Zürich, Switzerland.; ^5^ Stanford University, Stanford, CA, USA.; ^6^ University of Massachusetts Lowell, Lowell, MA, USA.

## Abstract

Wearable technology has shown notable promise for tracking human motion, offering valuable insights for fields ranging from biomechanics to healthcare. Traditional motion capture systems, however, are often bulky and disruptive, making them impractical for daily use. Advances in textile-based sensing offer a promising alternative, enabling seamless integration of air- and sweat-permeable sensors into everyday clothing. Here, a sensorized textile suit designed for unobtrusive full-body motion capture is presented. The suit is capable of accurately tracking complex movements without interfering with routine activities. This wearable, using an individual-customized network of fabric-based sensors, autonomously identifies and monitors movement angles and patterns, providing insights into physical range, activity frequency, and exertion levels. Language models are shown to interpret motion data into descriptive language, enhancing its potential for real-world applications. This sensorized textile suit and corresponding algorithms represent a step forward in accessible, continuous movement monitoring in the form of everyday clothing, opening avenues for studying human behavior and health in natural environments.

## Teaser

YSuit is a fully textile suit for accurate, customizable, washable, and comfortable anywhere full-body motion capture.

## INTRODUCTION

Wearable devices can provide rich physiological data that offers valuable insights into various bodily functions (*1*–*4*), such as heart rate (*5*), respiration rate (*6*), pulse oximetry (*7*), sweat levels (*8*), and body temperature (*9*). Some metrics, such as heart rate, can be influenced by both physical and mental states (*10*). Targeted sensing techniques can provide an opportunity to gain more precise behavioral insights (*11*, *12*). Specifically, analyzing full-body motion data from physical activities can uncover valuable information, spanning health, performance, entertainment, and social interactions (*13*).

Full-body motion data can be instrumental in health care by monitoring daily activity levels, identifying irregular movement patterns, and tailoring personalized treatment plans and interventions (*14*, *15*). In addition, beyond mere exercise tracking and goal setting for fitness and wellness (*16*), tracking detailed distributed body parts can enhance sport training programs, reduce injury risks, and improve performance analysis (*17*, *18*). Looking forward, the development of intuitive, user-friendly interfaces is crucial, especially in integrating gesture-based virtual reality systems and augmented reality applications (*19*, *20*). These interfaces can substantially enhance user experience and interaction, extending their applications in rehabilitation and the creation of assistive exoskeleton technologies for individuals with motor fatigue, disabilities, or mobility impairments (*21*–*23*). Broadly, full-body motion behavior data are also valuable for studies in psychology (*24*), biomechanics (*25*), neuroscience (*26*), and robotics (*27*), facilitating investigations into human movement behaviors (*28*), cognition (*29*), and social domains (*30*).

Human full-body motion data are commonly gathered through optical, electromagnetic (EM), and inertial measurement unit (IMU) systems (*31*). While optical systems (*32*) offer high accuracy, they require multiple cameras in a controlled laboratory environment and are hindered by labor-intensive marker labeling and occlusion issues (*33*). EM systems (*34*) offer greater flexibility but are prone to EM interference and pose dependence and still rely on external signal references (*35*). IMUs (*36*), which use gravity and inertia as references for motion, offer versatility across different environments but are vulnerable to transient mechanical vibrations and positional drift over time (*37*). In addition, rigidity remains a major problem for optical markers, EM sensors, and IMUs, which typically come in less user-friendly forms. The ideal solution would combine optical accuracy, EM flexibility, and IMU adaptability while being lightweight, comfortable, and capable of providing precise full-body motion data in any setting.

Soft strain sensors (*38*–*41*) offer a promising solution to achieve these goals. Typically composed of conductive composites and stretchable substrates, these sensors form an electromechanical structure to measure body stretch and joint bending. Commonly used conductive fillers include carbon nanotubes, graphene, metallic nanoparticles, and liquid metals (*42*), yet their skin compatibility needs further investigation (*43*). Silicone elastomers are often used as insulating host materials and highly stretchable substrates (*44*); however, concerns persist regarding thermophysiological comfort and skin health due to air and water-vapor permeability (*45*). Stretchable fabrics (*46*–*48*) are more breathable and comfortable, making them well-suited for wearable applications.

**Fig. 1.**
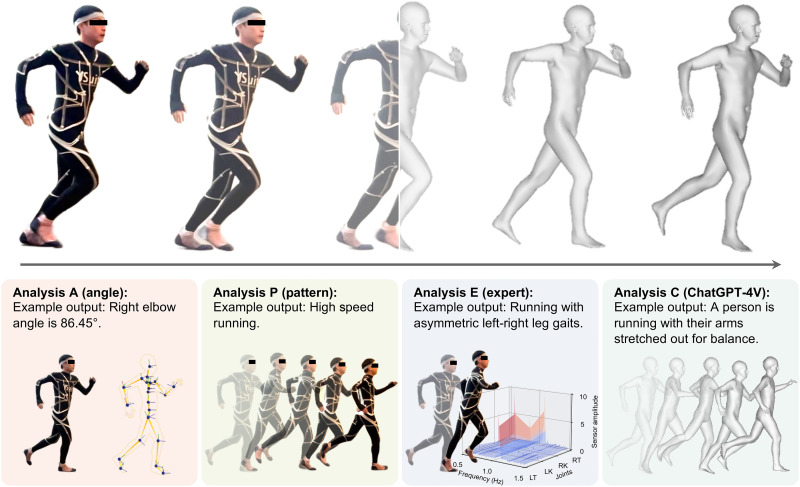
Overview. A user wears YSuit (**Left**) to capture their full-body motion data (**Right**) unobtrusively. The data are analyzed from multiple perspectives using different pipelines: frame-by-frame body joint angles (“A”), short-term movement pattern (“P”), human expertise (“E”), and large language model (“GPT-4V”). LK, left knee; RK, right knee; RT, right thigh; LT, left thigh.

To overcome these challenges, we introduce an innovative solution—a sensorized, washable, and breathable textile suit that provides a more unobtrusive, comfortable, and user-friendly approach to full-body motion tracking. Unlike existing methods, this suit enables continuous, real-time motion tracking in any environment, combining the ease of use with high accuracy, free from the constraints of rigid setups or bulky equipment. This wearable solution promises to revolutionize how motion data are captured, analyzed, and interacted with (as shown in Fig. 1), opening opportunities in health care, sports, and behavioral analysis.

Beginning with accuracy as our focal point, we recognize the significance of individual body shapes, leading us to propose a pipeline for customizing suits for individuals rather than adopting a one-size-fits-all approach. Our pipeline leverages vision techniques to reconstruct a three-dimensional (3D) human avatar, optimizing sensor placement based on the avatar’s structure and a comprehensive motion dataset covering full-body joint movement angles. This process results in a personalized suit design, equipped with 38 textile sensors that monitor 13 joints and capture 39 degrees of freedom (DoFs) across the entire body. To ensure wearer comfort, we use the same stretchable, breathable, washable, conductive fabric as the sensor unit to connect all sensors to a central data acquisition (DAQ) board. Portability is ensured by integrating the DAQ board with an onboard microprocessor and power supply, enabling real-time transmission of sensor data to a laptop via Bluetooth at 40 Hz, with 1 week of continuous use.

Our suit—which we refer to as the Yale Suit (YSuit)—is a full-body, wearable, anywhere-motion-capture garment. The YSuit demonstrates remarkable accuracy when compared to an optical motion capture system as ground truth. Through the development of supervised machine learning algorithms, we effectively map YSuit sensor readings to full-body joint angles provided by the optical system, achieving an average calibration accuracy of one degree for simple single-joint motions and two degrees for complex multijoint motions across all joints. Beyond assessing joint angles frame by frame, YSuit is used to identify motion patterns, offering insight into wearer behavior. These patterns encompass sequential motions, such as reaching for objects at different heights or varying walking speeds and step widths on flat, inclined, or declined paths. In addition, YSuit records 24/7 wearer behavior data to analyze activity patterns and joint movement throughout the day, highlighting potential fatigue-inducing activities. Recognizing the labor-intensive nature of analyzing extended sequential data, we further develop a pipeline that integrates large language models to interpret YSuit data. This approach streamlines data analysis, eliminates the need for manual data collection and labeling, and holds promise for developing large movement models capable of analyzing human behaviors at scale.

## RESULTS

### System introduction

Our portable YSuit, weighing 566 g, comprises a lightweight base suit (259 g) and a central DAQ unit (87 g). It houses 38 textile sensor units optimally positioned across the entire body to cover 13 body joints and 39 DoFs. These sensor units are interconnected via fabric-based conductive traces to the DAQ unit. The DAQ unit continuously transmits sensor readings from all 38 sensors over Bluetooth to a remote laptop at an average speed of 40 Hz, ensuring continuous data collection for over 1 week.

#### Textile sensor

**Fig. 2.**
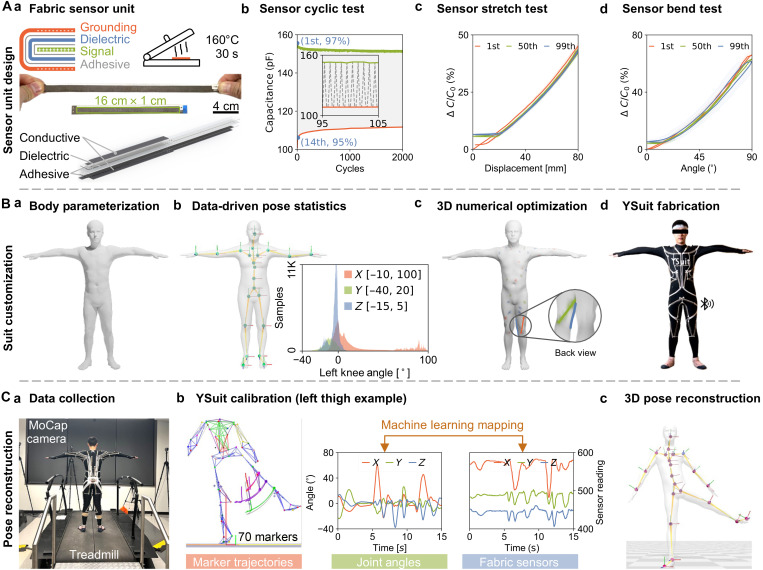
System introduction. (**A**) Textile sensor unit, including (a) working principle, manufacturing process, and a 3D exploded view, as well as evaluations under (b) cyclic, (c) stretching, and (d) bending tests. The symbols “+” and “–” in (a) indicate positive and negative charges, respectively. (**B**) Customization pipeline, featuring (a) the reconstructed digital human SMPL-X model, (b) AMASS pose statistics analysis of the showcased left knee, with rotation angles along the *X*, *Y*, and *Z* axes (represented in red, green, and blue, respectively), (c) numerical optimization of sensor placements for the left knee, and (d) the design and fabrication of the YSuit. (**C**) (a) Pose-capture setup, (b) machine learning–based calibration using labeled marker data from the optical motion capture system (O-MoCap), extracted joint angles, and YSuit sensor readings and (c) visualization of the reconstructed human pose.

The sensor unit is a five-layer textile-based capacitive sensor, featuring one internal conductive signal layer (silver plated 76% nylon and 24% elastic fiber) wrapped by two conductive grounding outer layers, separated by two nonconductive dielectric layers (80% nylon and 20% spandex), and bonded with thin films of breathable thermoplastic adhesives (Fig. 2Aa). The sensor unit measures 160 mm by 10 mm by 2.4 mm and is easily manufactured by pressing all laser cut materials simultaneously at 160°C for 30 s. The manufacturing method is compatible with rapid scale-up and mass production, facilitating future industrial-scale fabrication.

Under 2000 loading cycles with applied strain ranging from 0 to 50%, a representative sensor exhibits an increase in capacitance at 0% strain (base capacitance, indicated by the red line) and a decrease at 50% strain (maximum capacitance, indicated by the green line), as shown in Fig. 2Ab. The base capacitance drifts by 10.6%, while the maximum capacitance experiences a 3.1% drift more than 2000 cycles. The base capacitance drifted by 7.5% after the first cycle and reached 5.0% by the 14th cycle. This drift is defined as the percentage difference relative to the capacitance after the 2000th cycle. Over a 1-week period of nonuse, the sensor shows slight drift, but this drift can be reset by washing, as demonstrated in fig. S1 (B and C). Following manufacturing and washing, both the stretch test (Fig. 2Ac) and fix-end bend test (Fig. 2Ad) show low hysteresis during loading and unloading, demonstrating a homogeneous one-to-one correspondence between capacitance and displacement/angle, whereas the force-displacement displays comparatively larger hysteresis (fig. S1, Bd and Cd). The initial strain cycles induce plastic deformation in the sensors due to breaks in the thermoplastic adhesive, and subsequent cycles are stable. The sensor’s stability and washable nature for multiple uses hold promise for real-world applications.

#### YSuit customization pipeline with design and manufacturing

Considering variations in limb lengths and body shapes among individuals, we introduce a fully customizable suit design pipeline. We first reconstruct a 3D digital model of the user from an RGB video, as illustrated in Fig. 2Ba and fig. S2A. The model is based on SMPL-X parametric body models (*49*), which allow for easy animation and further analysis. Leveraging a large dataset of 3.97 million human daily poses (AMASS) (*50*), we conduct statistical analysis on the movement range of 13 joints with 39 DoFs throughout the entire body, showcased in Fig. 2Bb and fig. S2B. Based on each joint’s movement range, we simulate movements with the digital human and use grid search to determine the optimal direction with the maximum geodesic distance change over the skin surface, shown in Fig. 2Bc and fig. S2C. This simulation-based optimization procedure assists in deciding the direction and placement of the YSuit sensors, as shown in Fig. 2Bc and fig. S2Cb. Note that the current optimization procedure does not consider sensor overlapping, which should be avoided as increased thickness necessitates higher stretch force. Therefore, we rank the optimal directions and manually check for overlapping to choose them. Subsequently, we mark these optimal positions on a base suit (82% nylon and 18% spandex) for all 38 textile sensor units, as shown in fig. S2Cc.

We manufacture the textile sensors in a batch of 50 with a factory sensor reading variance of around 2%. The sensors undergo 100 precycles under 50% strain on an automatic testbed to eliminate plastic deformation, followed by washing and drying for assembly, as depicted in fig. S2D. All sensors are then heat-pressed onto the base suit at optimal locations. Conductive fabric traces (4 mm wide), the same material with a surface resistivity of 0.5 Ω/□ as the sensor themselves, interconnect distributed fabric sensors to a central DAQ unit. These traces form parasitic capacitors that increase sensor capacitance under no strain (fig. S1Ad) and decrease under cyclic strain due to increased distance between traces (fig. S1E). The DAQ unit has a form factor of 66 mm by 42 mm by 28 mm and weighs 87 g. It can access 48 capacitive sensors in serial at 40 Hz and is powered by a 1000-mAh battery, allowing for over 1 week of data collection. Equipped with an onboard microprocessor (Adafruit Feather 32u4 Bluefruit LE), it transmits all sensor readings to a remote laptop via Bluetooth. All materials used in YSuit are fabric, elastic, and breathable, ensuring a comfortable user experience. The customized suit is lightweight and portable, facilitating long-term human motion tracking.

#### Data processing pipeline

YSuit introduces a data type that physically measures local skin stretch induced by joint movements, distinguishing it from optical, EM, and IMU systems that rely on global coordinates (stationary cameras, base transmitters, and gravity). To assess YSuit’s accuracy in capturing whole joint angles, we compare it to an optical motion capture system (O-MoCap) as the ground truth, which is considered the gold standard (*31*).

The motion capture system collects 3D kinematics using eight cameras (100 Hz, Motion Analysis Corporation, Santa Rosa, CA, USA) that track 70 reflective markers attached to the user’s body (Fig. 2Ca). Labeling markers for sequential motions is labor-intensive due to unavoidable visual occlusions and pseudo-recognized markers from the surroundings, necessitating postprocessing of marker kinematics to extract joint angles using a visual 3D software (C-Motion Inc., Germantown, MD, USA), as shown in Fig. 2Cb. This process includes data filtering with a fourth-order Butterworth low-pass filter (cutoff frequency of 10 Hz) to eliminate marker vibration caused by inertia effects. Later on, we develop supervised machine learning algorithms (see Materials and Methods) to regress YSuit sensor data to the joint angles from the O-MoCap, as shown in Fig. 2Cb. Ultimately, we reconstruct and visualize the human pose based on the regressed joint angles from YSuit sensor data, as depicted in Fig. 2Cc.

### Accuracy evaluation

Human activities and motions vary widely, from slow single-joint movements to fast multijoint actions. Here, we assess the accuracy of the YSuit system across this diversity of motion. YSuit uses a machine learning approach, using a trained model to predict angles of 11 joints with 33 DoFs based on raw measurements from 38 textile sensors. Note that YSuit is designed for 13 joints with 39 DoFs; however, the optical motion capture system can only access 11 joints. Both YSuit and O-MoCap systems collect data while the wearer performs various motions. The collected data are randomly split into training, validation, and test datasets with a ratio of 3:1:1. During runtime, a multilayer perceptron (MLP) structured machine learning model (five layers with 100 units each), trained on the training dataset and fine-tuned using the validation dataset, maps raw YSuit textile sensor values to joint angles. For evaluation, we compare the predicted joint angles with those obtained from O-MoCap in the test dataset. Details are in Materials and Methods.

#### Single-joint movement

**Fig. 3.**
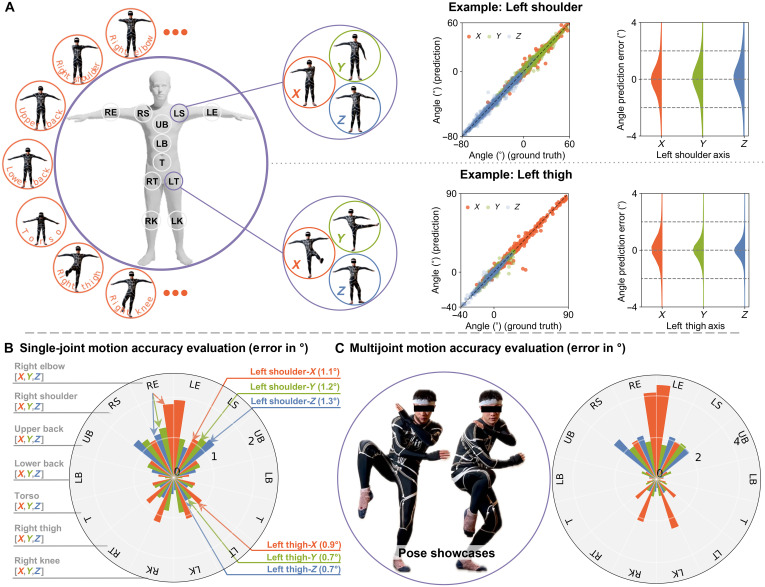
Accuracy evaluation. (**A**) Demonstration of single-joint movements (left) and machine learning–driven calibrated angle prediction (P) compared to ground truth (GT) for *X*, *Y*, and *Z* orientations of left shoulder (LS) and left thigh (LT) in the middle. Statistical evaluation of the angle prediction error is shown on the right. (**B**) Quantitative evaluation (left) displayed as a polar bar plot, illustrating the spatial distribution of accuracy over the human body. (**C**) Visualization of multijoint motion (left) with spatial accuracy distribution for multijoint motion calibration (right). LE, left elbow; LS, left shoulder; RE, right elbow; RS, right shoulder; UB, upper back; LB, lower back; T, torso.

The wearer executes a sequence of motions, rotating 11 body joints individually, as depicted in Fig. 3A. Because of the proximity of the shoulder and collar joints, rotations are performed along 11 body joints instead of 13. In addition, keeping other joints absolutely stationary while executing single-joint movements is challenging. Therefore, the wearer primarily focuses on repeating rotations of each joint in the *X*, *Y*, and *Z* directions five times, with no restrictions on other joint movements. The middle section of Fig. 3A showcases the angle predictions of the left shoulder and left thigh, which closely match the O-MoCap angle measurements (ground truth). The prediction errors are predominantly within 1°. Quantitative evaluations of all joint angle predictions are summarized in Fig. 3B, where most of the mean joint angle errors fall within 1°. Higher errors are observed for the left elbow, right elbow, left knee, and right knee, hypothetically due to long interconnecting wires between the sensor and the DAQ. Furthermore, upper limb movements exhibit higher errors compared to lower limbs, likely due to their greater range of motion and movement across three dimensions. We also observe that the sensor network, consisting of 38 sensors, outperforms the single-sensor-to-single-joint mapping method, as shown in fig. S 11A and detailed in the “Sensing network” section in the Supplementary Text. Incorporating more sensors to predict a single joint enhances accuracy, as joint movements are always coupled, and the increased number of sensors helps mitigate data ambiguity and noise. The theoretical explanation is provided in the “Theory accuracy reference” section in the Supplementary Text.

#### Short-term application

**Fig. 4.**
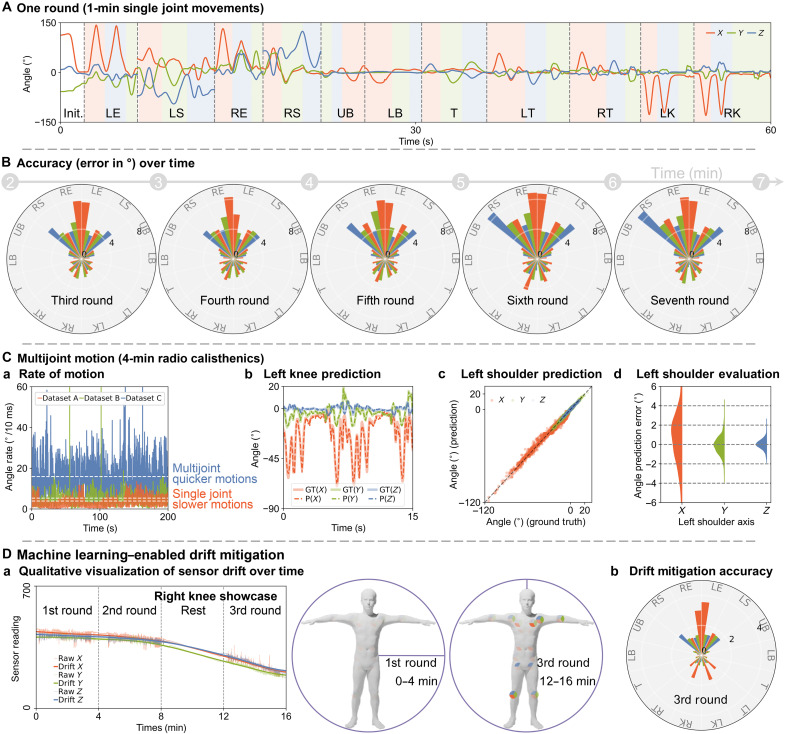
Comprehensive accuracy evaluation. (**A**) Single-joint motion angle changes during a 1-min cycle. (**B**) YSuit prediction accuracy trends over five subsequent movement cycles. (**C**) Multijoint motion analysis of dataset C (Fig. 3C) compared to datasets A and B based on angle change rate. Joint angle prediction showcases include: (b) left knee over time, (c) left shoulder movement range, and (d) left shoulder statistical evaluation. (**D**) Sensor drift visualization during sequential rounds of multijoint motion: (a) drift over time and (b) spatial accuracy distribution after drift mitigation.

The calibration model is accurate (Fig. 3A), indicating that the machine learning model can generalize knowledge to the data within its distribution. We therefore assess YSuit’s short-term accuracy for practical applications. We instruct the wearer to perform 1-min single-joint movements for all joints in three orientations (Fig. 4A), repeated seven times. The pose motions resemble those in Fig. 3A with minor deviations in angles and speed. Using the first two rounds for training an MLP model, we predict angles for the subsequent five rounds. Quantitative results (Fig. 4B) reveal a fourfold increase in error compared to previous assessments. Lower limb accuracy surpasses that of upper limbs, with elbows exhibiting the most significant discrepancies presumably due to extended wire connections and potential O-MoCap inaccuracies. Moreover, a slight increase in error over time suggests possible translation of sensors along the surface of the body.

#### Multijoint movement

**Fig. 5.**
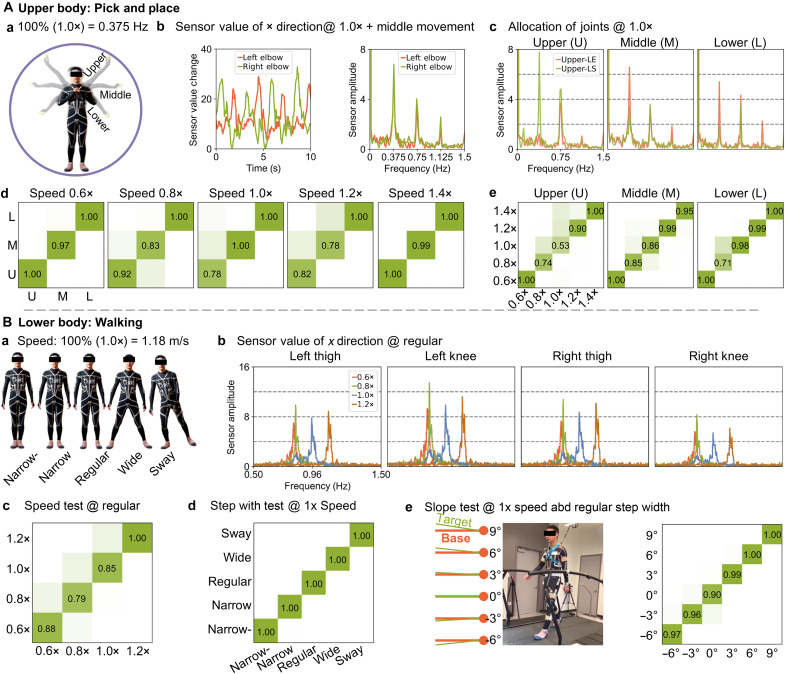
Pattern recognition. (**A**) (a) Upper body pick-and-place poses. (b) Sensor readings visualized in both time and frequency domains. (c) Joint allocations for upper, middle, and lower movements visualized in the frequency domain. Movement patterns are classified based on (d) speeds and (e) heights. A classification accuracy of 1.00 represents 100% accuracy. (**B**) (a) Lower body walking gaits. (b) Joint allocations for different speeds visualized in the frequency domain. Patterns are classified based on (c) speeds, (d) step widths, and (e) slopes.

Beyond simple single-joint motions, we explore YSuit’s potential for complex multiple-joint motions, as showcased in Fig. 3C. We instruct the user to perform a 4-min multijoint radio calisthenics motion (*51*). Quantitative analysis (Fig. 4Ca) compares the all-summed-angle change rate between single-joint slower motion (Figs. 3A: 3.9°/10 ms and 4A: 5.9°/10 ms) and multijoint quicker motions (Fig. 3C: 15.9°/10 ms); all are mean values. The all-summed-angle change rate is defined as the total angular changes of all body joints over a 10-ms time interval. The trained calibration model exhibits a twofold error compared to the single-joint motion model with all other observations remaining consistent, as shown in Fig. 3C. Supplementary comparisons (Fig. 5 and fig. S8) indicate that this increase in error is driven primarily by the added motion-pattern complexity of multijoint movements rather than by the higher execution speed alone. Compared to knee joint angle predictions (Fig. 4Cb), shoulder predictions exhibit higher errors in the *X* direction (Fig. 4 C, c and d), with a consistent offset of 2°. This is likely due to the sensor’s translation along the body surface. Despite this translation aspect, the YSuit can still capture human motions accurately, with accuracy only being affected twofold even as the activity becomes four times more complex.

#### Multijoint drift mitigation

The sensors are susceptible to drift due to physiological reactions, such as sweat, and environmental conditions, such as humidity (*48*). We therefore implement a drift mitigation strategy.

To induce sensor drift, we instruct the user to repeat the radio calisthenics for another two rounds, with a four-minute pause in between. As shown in Fig. 4Da and fig. S5, while the sensor readings correspond to joint movements, they also exhibit continuous drift over 16-min. The wearer reported sweating during this procedure, prompting us to conduct an ablation test by soaking five textile sensor units in a simplified solution comprising 9 g of NaCl and 1000 ml of deionized (DI) water. The mean value of the base capacitance is 100.6 pF initially, 60.4 nF after soaking, and 93.9 pF after drying for 24 hours at 60°C. The wet solution changes the capacitance by several orders of magnitude.

We further investigate the drift distribution of all sensors over the body over time. As shown in Fig. 4Da and fig. S6, the drift first occurs near the armpits and knees and spreads across the whole body, which coincides with the reported distribution of sweat (*52*). This indicates that the sensor unit could be used to monitor sweat, a possibility we leave for future exploration. Despite this drift, our data processing pipeline is still capable of calibrating the sensor with comparable accuracy under this drift condition, as shown in Fig. 4Db.

### Pattern recognition

The previous frame-by-frame pose reconstruction is crucial for certain applications. In addition, we propose two alternative approaches to analyzing human behavior. Human motions consist of sequences of poses that characterize specific activities. We use our upper limb joints for reaching, grasping, and placing objects and our lower limbs for walking with varying step widths and over different terrains. Understanding these patterns over certain periods enables us to discern human activities and enhance performance. Moreover, in addition to frame-by-frame pose reconstruction, analyzing raw sensor values in the frequency domain provides temporal information about frequency and magnitude. This approach allows us to extract data on speed, spatial distribution of joint movements across the entire body, and other relevant information directly. Furthermore, the drift effect from sweating or sensor placement can be negligible when we only consider the speed and relative movement range of whole body joints.

#### Upper-body pick and place

We instruct the wearer to hold a tennis ball in front of their chest (center position) and reach to left and right sides iteratively at specific height levels of upper, middle, and lower positions, as depicted in Fig. 5Aa and figs. S7Aa and S8A. To control the speeds (0.6×, 0.8×, 1.0×, 1.2×, and 1.4×) of these motions, we use the Google Metronome software, with the base speed (1.0×) set to 90 beats per minute (BPM). The polar plot in fig. S8A top-left corner shows the frame-by-frame prediction accuracy for the motions. The lower limbs exhibit precise tracking, while the upper body remains within one degree of accuracy, except for the elbows, which exhibit an error of over three degrees. As showcased in Fig. 5Ab, periodic sensor readings are complex in the time domain but clean in the frequency domain, allowing us to extract movement frequency and amplitude and eliminate the stationary pose offset (T-Pose or A-Pose). Qualitative analysis in Fig. 5Ac and fig. S7Ba shows that the wearer uses shoulders more than elbows for upper height, elbows predominantly for middle height, and elbows slightly more than shoulders for lower height, at all speeds.

In addition, we conduct quantitative evaluations by classifying motions based on speed and height. Given that motions occur sequentially, we use a long short-term memory (LSTM) network structure (*53*) to capture time dependence. The LSTM has a look-back parameter, defining how many past data points influence the current prediction. At controlled speeds [Fig. 5A (iv)] and heights [Fig. 5A(v)], the motion heights and speeds can be accurately identified: (i) Heights are precisely classified at speeds of 0.6× and 1.4×, with accuracy decreasing at speeds of 0.8×, 1.0×, and 1.2×. (ii) Speeds are accurately classified for middle and lower heights but slightly less for upper heights. Misclassifications may result from similarities in movement behavior over the sequence time window; the LSTM’s time window parameter (1 step/10 ms used here) affects classification (fig. S9B). When all recorded data with five different speeds and three heights are shuffled into 15 classes, the trained LSTM model (with one look-back step) can correctly identify all classes (figs. S7A and S8A).

#### Lower-body walking

Similar to the upper limb pattern recognition analysis, we analyze lower limb movements by instructing the wearer to walk on a treadmill (M-Gait, Motek, Netherlands) at various speeds (0.6×, 0.8×, and 1.0× equivalent to 1.18 m/s and 1.2×), step widths (narrow-, narrow, regular, wide, and sway), and slopes (−6°, −3°, 0°, 3°, 6°, and 9°). The accuracy study in fig. S8B polar plot reveals precise frame-by-frame reconstruction within all joints with an error margin of two degrees. Qualitative frequency analysis in Fig. 5Bb and fig. S10B shows that: (i) The wearer increases the iteration frequencies of lower limb movements to catch up with increased speed overall, with a specific increase in joint angles at 0.8× speed. (ii) Besides asymmetric gait amplitudes from left to right, the wearer redistributes thigh and knee angles and frequencies to adapt to changes in step widths (e.g., bending the hips more with low speed for sway and bending the knees more with faster speed for narrow- and narrow, with the wide step width as an exception). (iii) For declined slopes, the wearer tends to use smaller joint-angle gaits and faster speed, while for inclined slopes, the wearer tends to slow down the speed but increase hip angles and reduce knee angles. Quantitative evaluations of the pattern classification task demonstrate that walking speed levels can be classified with ~80% accuracy or higher (Fig. 5Bc), 100% accuracy for step widths (Fig. 5Bd), and above 90% accuracy for slopes (Fig. 5Be). All 15 classes of shuffled walking gaits can be correctly classified, as depicted in fig. S8B.

### Applications

In addition to frame-by-frame pose reconstruction, we offer multidimensional analysis strategies for analyzing human motions based on YSuit data. The YSuit demonstrates accuracy (within 2°, referring to the O-MoCap system as ground truth) and can be used for precise pattern classification. Time-frequency domain analysis of the YSuit data provides multifaceted information on human motion. Now, we explore the potential of YSuit for large time-scale applications.

#### 24-hour behavior analysis

**Fig. 6.**
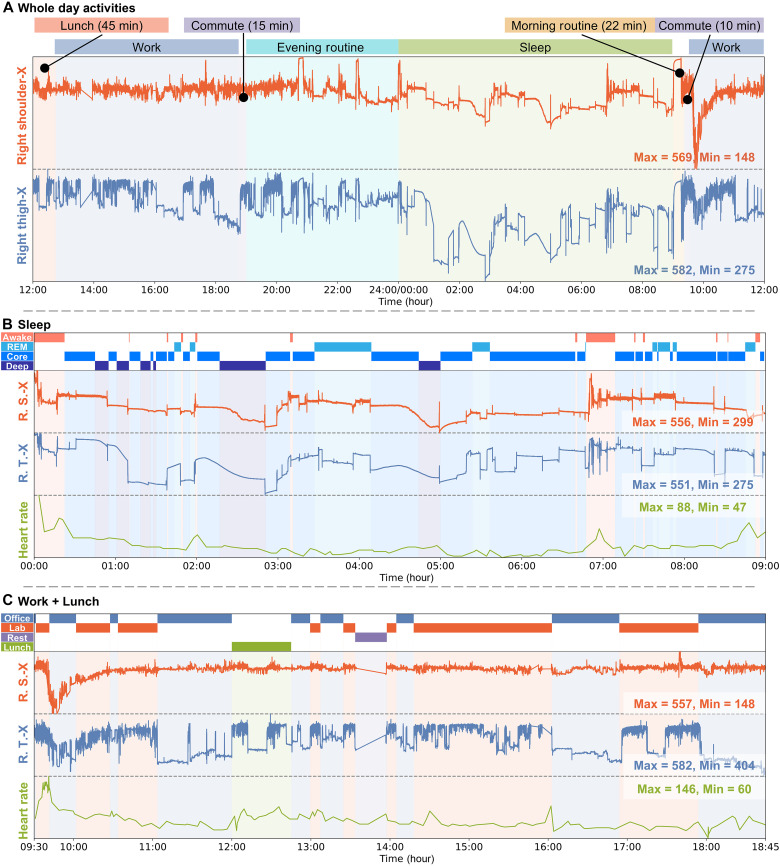
24-hour activities with detailed categories. (**A**) Normalized sensor readings of right shoulder orientation (R.S.-X) and right thigh orientation (R.T.-X) in the *X* direction during various whole-day activities, including lunch, work, commute, and evening and morning routines. The normalization is computed as $S_{i}=\left[S_{i}-\text{min}\left(\forall S_{i}\right)\right]/\left[\text{max}\left(\forall S_{i}\right)-\text{min}\left(\forall S_{i}\right)\right]$. (**B**) Normalized right shoulder and right thigh movements, along with heart rate, during sleep. Sleep phases are categorized into awake, rapid eye movement (REM), core, and deep stages. (**C**) Normalized right shoulder and right thigh movements, as well as heart rate, during work and lunch. Activities are further categorized into office work, laboratory work, rest, and lunch.

Pose reconstruction and pattern classification typically require data collection in confined laboratory spaces and extensive manual labeling efforts. Given the diverse nature of human motions, YSuit needs to extend beyond the lab environment. To achieve this, we initially use YSuit for continuous 24-hour monitoring of the wearer’s activities. This allows us to capture the wearer’s behavior throughout the day, including sleep, morning and evening routines, meals, commute, and work, as illustrated in Fig. 6A. By analyzing the signals from the right shoulder (RS) and right thigh (RT) sensors over the entire day, we qualitatively visualize how the wearer uses their upper and lower limbs. Observations reveal static or quasi-static poses during sleep, increased upper and lower limb movements during the morning routine, decreased activity during the evening routine, and steady coupled or iterative upper and lower limb movements during commute, work, and lunch.

We zoom in on the phases of sleep (Fig. 6B) and work + lunch (Fig. 6C). Using an Apple Watch Series 9 as a simplified reference for heart rate and sleep phases, we observe frequent limb changes during “Awake,” quasi-static poses during “REM,” steady poses with occasional changes during “Core,” and steadily increased bending angles during “Deep,” with most transitions between sleep phases accompanied by a pose change. The observation of increased bending angles during Deep might be due to decreased heart rate and decreased body temperature, which could affect the sensor readings, warranting further investigation in future work. During work and lunch, the wearer used upper limbs more for office work and lunch while sitting, with lower limbs used more frequently and intensely for commuting and laboratory work. All these raw sensor readings provide qualitative information about human behaviors, opening up possibilities for wide applications reliant on motion data.

#### Fatigue modeling

**Fig. 7.**
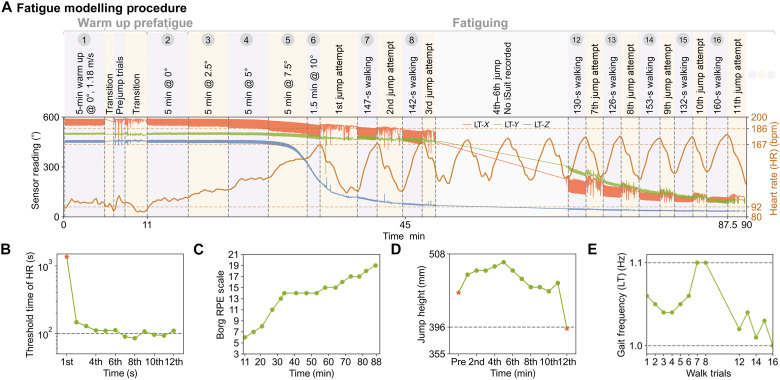
Fatigue modeling. Introduction to (**A**) the fatigue modeling procedure and monitoring of (**B**) heart rate, (**C**) subjective Borg RPE scale, (**D**) jump height, and (**E**) changes in gait frequency.

Endurance activities such as long-distance walking or hiking can lead to motor fatigue over time. To model this fatigue, we follow the experiment protocol in (*21*) by having the wearer walk on a treadmill with gradually increasing inclines (Fig. 7A). During the walking trials, we record jump heights (Fig. 7D) and subjective difficulty using the Borg rating of perceived exertion (RPE) (Fig. 7C), after the wearer’s heart rate exceeds 85% of the maximum age-predicted value (Fig. 7B). The fatigue procedure concludes once a reduced jump height of 20% is observed. During the fatigue procedure, we observe an increased heart rate corresponding to increased inclination and faster heart rate elevation (Fig. 7A), increased difficulty (Fig. 7C), and slightly reduced jump height (Fig. 7D) over the progressing trials. Based on the YSuit data, the wearer initially adapted to the procedure with decreased gait frequencies and steadily increased gait frequencies as the treadmill incline angle increased. However, as the fatigue procedure continued, the wearer reduced their gait frequencies with less stability (Fig. 7E and fig. S11A). Moreover, in conjunction with the wearer’s report of sweating during the procedure, we observed sensor unit drift coinciding with the increase in heart rate. The drift (fig. S19B) initially occurred near the armpits and knees and then propagated to the upper and lower limbs, eventually covering the entire body, including the abdomen and back. These observations are qualitative, indicating the potential for our YSuit to be used in real-world human behavior analysis applications and opening doors for further exploration.

#### Large language model (GPT-4V)

**Fig. 8.**
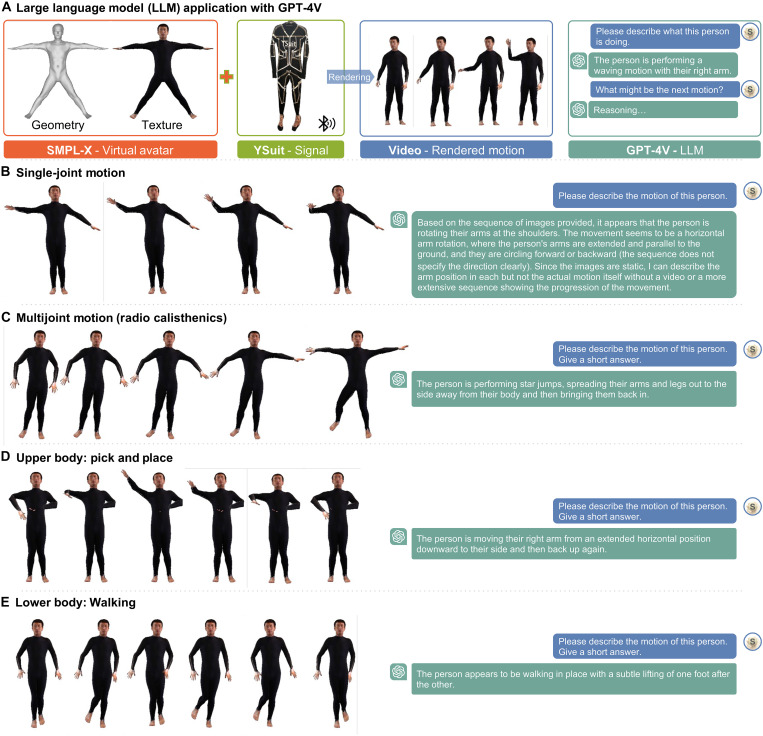
Motion descriptions with GPT-4V. (**A**) Pipeline for building large motion models to analyze individual behavior. (**B** to **E**) Examples of single-joint motion, multijoint motion, and upper- and lower-body motion, respectively. The images are rendered from the wearer’s digital avatar using poses estimated from the YSuit signal. The conversations display text queries and responses generated by GPT-4V.

Equipped with YSuit signals or joint information predicted from these signals, we aim to analyze and understand holistic behavior. While manual checking of motions is an option, it is labor-intensive, is time-consuming, and may lack accuracy and precision in describing motions. Fortunately, large foundation models, such as GPT-4V, which have been trained on massive text and image datasets, excel at describing images or videos. However, because these models only accept text or image inputs, we have developed a pipeline that converts YSuit signals into motion videos using estimated SMPL-X poses and the reconstructed body model of the wearer. These videos can then be used as inputs for GPT-4V to analyze behavior in textual form (Fig. 8A). GPT-4V provides detailed descriptions of motion, both frame-by-frame and sequence-by-sequence. Examples are shown in Fig. 8. This method of analyzing motion not only enhances our understanding of human behaviors but can also facilitate the collection of long-term motion data. These data can be instrumental in developing more sophisticated large motion models, with notable potential benefits for health care, performance enhancement, metaverse applications, and social interaction.

## DISCUSSION

Our key contribution is the YSuit, a customizable, comfortable, portable, washable, and affordable tool that accurately reconstructs human motion, enabling precise capture of human pose and movement. We offer various methods for interpreting YSuit data, allowing for the extraction of multifaceted information to analyze human pose, motion, and behavior. We showcase YSuit’s potential for large-scale applications in tracking long-term human daily activities, evaluating endurance, and building large motion models for behavior understanding.

YSuit offers a streamlined pipeline for customization based on individual body shape and size, while our simplified manufacturing process enables factory-level scalability.

Although customization may initially appear more costly compared to optical motion capture systems, EM systems, or IMUs, it could be more practical for real-world applications. Traditional systems require optical markers, EM devices, or IMUs to be repeatedly and precisely attached to a base suit before each use—resulting in high labor costs. In contrast, our solution requires only a one-time customization, significantly reducing long-term operational overhead.

Future work will focus on optimizing the YSuit design, conducting user studies for specific applications, and building large-scale motion models. In terms of design optimization, we observed gradual accuracy degradation during use, primarily due to sensor unit performance degradation and sensor position shifts caused by relative movement between the sensors and the human body.

The YSuit fabrics, composed of commonly used durable materials such as nylon and spandex (see Materials and Methods), remained stable throughout approximately 3 months of study use. Quantitatively, our 2000-cycle test demonstrated reliable performance (Fig. 2, Ab), which is also supported by previously reported cyclic testing up to 5000 cycles [$\Delta C/C_{0}\approx 0.45\%$ at 60% strain; (*48*)].

While stress relaxation and fiber rearrangements within the fabric contribute to this drift issue over time, our evaluation over a 1-week period showed that accuracy is retained after regular washing and drying (fig. S1Cb). The long-term drift characterized by a decrease in sensor capacitance under strain (fig. S1B) may be attributed to factors such as humidity absorption, stress relaxation, and ionic redistribution. Our previous study (*48*) demonstrated reduced sensitivity under elevated humidity levels, supporting the hypothesis that moisture absorption suppresses sensor response. In addition, postcyclic testing reveals an increase in baseline capacitance (fig. S1, Bb and Cb), suggesting the presence of stress relaxation and fiber rearrangements within the sensor material. While ionic redistribution remains a plausible mechanism, it has yet to be experimentally verified and warrants future investigation. Based on the current evidence, humidity absorption appears to be the primary contributor to the observed capacitance drift. Future material optimization may reduce the sensor’s susceptibility to humidity absorption.

To mitigate positional shift between the suit and human skin, several optimization strategies may be pursued, including refined geometric designs to constrain sensor positional shifts, machine learning–based compensation algorithms that account for material aging effects, and multisensor fusion techniques to enhance robustness and precision. In addition, practical design factors such as interconnection length, parasitic coupling, and multijoint activation should be considered, as they can influence performance during long-duration or mobile use. Beyond geometric refinement, another approach could be the incorporation of an ultrathin, breathable epidermal adhesive layer between the skin and the suit, as demonstrated by Zhang *et al.* (*5*), which can effectively minimizes sensor position shifts but would decrease the garment-like nature of the Ysuit. Furthermore, a sim-to-real transfer learning pipeline—where sensor data, particularly the effects of positional shift, are simulated on a biomechanical model of the user—could further improve accuracy from an algorithm perspective.

Additional factors contributing to drift may include long-term changes in the user’s body shape (e.g., due to weight fluctuations) and sweat accumulation during high-intensity movement. These issues could be mitigated through simplified recalibration methods—for instance, a preuse calibration procedure using a smartphone camera with SMPL-X techniques and a set of guided motions could offer a practical and user-friendly solution. While the present study validates the pipeline on a single subject, future work will include multisubject testing to evaluate cross-subject generalizability and robustness to variations in anatomy and movement biomechanics. We are also exploring a sim-to-real transfer approach that would use biomechanical models to simulate sensor data for new users, potentially enabling rapid adaptation to different body sizes and shapes. Another promising direction is enabling users to wear the YSuit during everyday activities, allowing for the collection of large-scale behavioral datasets. These datasets could be used to train generalizable motion models, unlocking a wide range of applications, from human behavior analysis to health monitoring and disease prediction.

We envision the advancements presented here paving the way for widespread adoption, ultimately enhancing health, fitness, and overall quality of life in society.

## MATERIALS AND METHODS

### Textile sensor unit

The sensor unit is a five-layer textile-based capacitive sensor, featuring one internal conductive signal layer (silver plated 76% nylon and 24% elastic fiber, purchased from FilterEMF) wrapped by two conductive grounding outer layers, separated by two nonconductive dielectric layers (80% nylon and 20% spandex, purchased from Amazon), and bonded with thin films of breathable thermoplastic fabric adhesives (3410 Sewfree Tape, purchased from Bemis Associates Inc.). According to the parallel-plate capacitance model, reducing the thickness of the dielectric layer increases the baseline capacitance and improves sensitivity to pressure variations induced by stretching or bending. In our implementation, we used a consistent thickness across all sensors to ensure uniform performance, but we acknowledge that varying the thickness could further tune sensor behavior. The sensor unit has a form factor of 160 mm by 10 mm by 2.4 mm designed in SolidWorks 2020. All fabric materials were laser cut into the geometries using a Universal laser cutter (Universal Laser Cutter VLS2.30DT, purchased from Universal Laser Systems Inc.). The laser cutter settings were 50, 50, and 100% of full speed intensity and 70, 50, and 90% of full power intensity for conductive fabric, nonconductive fabric, and adhesive film, respectively. The final sensor unit was manufactured by heat-pressing (Tusy Heat Press Machine, purchased from Amazon) all stacked laser-cut materials simultaneously at 160°C for 30 s. All sensors fabricated using the thermoplastic adhesive exhibited no delamination during cyclic mechanical testing, multiple washing cycles, or over the course of 3 months of continuous data collection.

### Characterization of textile sensor unit and fabric trace

An LCR meter (E4980AL, purchased from Keysight Technologies) was used to measure capacitances (Cs) of textile sensor units with a measurement setting of 1-V exciting voltage and 1-kHz exciting frequency. A material stretch-testing system (Instron 3345 with a 50-N load cell as shown in fig. S1Ba, purchased from Illinois Tool Works Inc.) was used to quantify sensor units’ force-displacement-capacitance correlation with a rate of 5 mm/s and a maximum displacement of 80 mm equaling to 50% of 160 mm. A customized material bend-testing system was built as shown in fig. S1Da (one servo motor-Dynamixel Mx-106 T purchased from Robotis Inc. and two artificial limb parts 3D printed by Original Prusa i3 MK3S+) to quantify the sensor units’ angle-capacitance correlation with a rotation speed of 38°/s and a maximum angle of 90°. Each data point in all plots has one mean value and one SD value evaluated on five textile sensor unit samples. The characterization of fabric trace was conducted using the stretch-testing system at 50% strain. The washing test of the sensor units was conducted at 25°C by diluting 3 ml of a commercial neutral detergent (TexCare #A289-L, purchased from FilterEMF) into 1000 ml of DI water (PURELAB Flex 3 System, purchased from Evoqua Water Technologies LLC) at a pH 9.7, and subsequent continuous stirring for 60 min as shown in fig. S1Ca. After this, the sensor units were stirred in pure DI water at a pH 6.1 for 60 min and dried 24 hours in an oven at 60°C. For the simplified sweat test, textile sensor units were soaked in a simplified solution comprising 9 g of sodium chloride (S9888, purchased from Sigma-Aldrich) and 1000 ml of DI water for 60 min and subsequently dried 24 hours in an oven at 60°C.

### Customization pipeline

The pipeline starts with a digital registration of the wearer (fig. S2A) from a video of the wearer (fig. S2Aa) to a digital 3D avatar (fig. S2Ac). First, an iPhone 13 Pro was used to record a video (60 fps) of the wearer with arms straight out (T-pose) and slowly completing a full rotation. The recorded video was then processed to segment foreground image of the wearer. A universal SMPL-X modeling procedure (*49*) was used to reconstruct the final digital 3D avatar for the wearer considering geometry and texture (color), minimizing the L1 loss between the rendered colored and posed body and the segmented foreground image (fig. S2 Ab). The pipeline continues with a joint movement range analysis for 13 joints, 39 DoF based on 3,971,045 poses in the AMASS dataset (*50*). All 39 joint movement range were decided on the basis of their histogram (2000 bins over a range from −180° to 180°), as shown in fig. S2B. Based on each joint movement range, we placed virtual sensors on the digital human and simulated the stretching movement of these sensors by calculating the geodesic distance change over the skin surface. We conducted a grid search for all possible sensor placement positions and determined the optimal locations by ranking them according to the maximum relative stretch. The current optimization procedure did not consider sensor overlapping, which should be avoided as increased thickness necessitates higher stretch force. Therefore, we ranked the optimal directions and manually checked to choose them. Subsequently, we marked these optimal positions on a base suit (82% nylon and 18% spandex, purchased from Amazon) for all 38 textile sensor units, as shown in fig. S2Cc.

### YSuit integration pipeline

We manufactured textile sensor units in a batch of 50 (geometry design → materials preparation → laser cutting materials → heat press). The sensors underwent 100 precycles under 50% strain on an automatic testbed (Instron at a rate of 5 mm/s) to eliminate plastic deformation, followed by washing (3‰ TexCure solution in DI water with a pH 9.7, 60-min stirring), postwashing (DI water with a pH 6.1, 60-min stirring), and drying (24 hours in an oven at 60°C), as depicted in fig. S2Db. All sensors were then heat-pressed (160°C, 30 s) onto the base suit at optimal locations. Laser-cut 4-mm-wide conductive fabric traces were heat-pressed on the base suit with the thermoplastic adhesive film in between to connect the sensors to a DAQ unit. The DAQ unit has a form factor of 66 mm by 42 mm by 28 mm and weighs 87 g. It includes an onboard microprocessor (Adafruit Feather 32u4 Bluefruit LE, purchased from Adafruit) that communicates with four 12-channel capacitive touch sensor breakout boards (Adafruit MPR121, purchased from Adafruit) via I^2^C communication protocol. It is powered by a 1000-mAh battery and transmits 38 capacitive sensor readings in serial to a remote laptop (Apple MacBook Pro M2) via Bluetooth communication protocol at 40 Hz (fig. S2Dd).

### Data collection for accuracy study

For the accuracy study and the pattern recognition study, we simultaneously collected YSuit data and 3D kinematics using an eight-camera video system (100 Hz, Motion Analysis Corporation, Santa Rosa, CA, USA), with 70 reflective markers attached to the user’s whole body (Fig. 2Ca). For the accuracy study in Fig. 3A, we instructed the wearer to repeatedly rotate single joint five times along *X*, *Y*, and *Z* directions in serial, following the joint order of left elbow (LE), left shoulder (LS), right elbow (RE), right shoulder (RS), upper back (UB), lower back (LB), torso (T), left thigh (LT), right thigh (RT), left knee (LK), and right knee (RK). We name this as the single joint motion dataset (dataset A). For the accuracy study in Fig. 4A, we instructed the wearer to rotate single joint only once along *X*, *Y*, and *Z* directions in serial, following the same joint order. This took around 1 min, which we call one round of 1-min single joint movement (dataset B). We instructed the wearer to implement seven rounds in serial with no break. For the accuracy study in Figs. 3C and 4C, we instructed the wearer to implement a 4-min radio calisthenics (https://www.youtube.com/watch?v=GuMIomW9wwg). We name this as the multijoint motion dataset (dataset C). For the accuracy study in Fig. 4D, we instructed the wear to repeat the 4-min radio calisthenics twice with a 4-min break in between. We name this the drift mitigation dataset (dataset D).

### Data collection for pattern recognition study

For the pattern recognition study in Fig. 5A and figs. S7Aa and S8A, we instructed the wearer to hold a tennis ball in front of the chest (centre position) and reach to left and right sides iteratively at specific height levels of upper, middle, and lower positions, as depicted in Fig. 5A and figs. S7Aa and S8A. To control the speeds (0.6×, 0.8×, 1.0×, 1.2×, and 1.4×) of these motions, we used the Google Metronome software, with the base speed (1.0×) set to 90 BPM. The wearer was instructed to switch between motions every 1 min. We name this as the upper-body pick-and-place dataset. For the pattern recognition study in Fig. 5B and figs. S8B and S10Aa, we instruct the wearer to walk on a treadmill (M-Gait, Motek, Netherlands) at various speeds (0.6×, 0.8×, and 1.0× equivalent to 1.18 m/s and 1.2×), step widths (narrow-, narrow, regular, wide, and sway), and slops (−6°, −3°, 0°, 3°, 6°, and 9°). The wearer was instructed to switch between motions every 1 min. We name this as the lower-body walking dataset.

### Data collection for application study

For the application study in Fig. 6, the wearer donned the YSuit and a wrist smart watch (Apple Watch Series 9) for 24 hours. The watch recorded heart rate, body temperature, steps, and sleep stages. We name this as the 24 hours behavior understanding dataset. For the application study in Fig. 7, we recorded YSuit data, jump height (Vertec vertical jump tester, JumpUSA), Borg RPE, and heart rate (EQ02 + LifeMonitor, purchased from Equivital). The wearer was first given time to familiarize themselves to walking on a treadmill (M-Gait, Motek, Netherlands) at 1.18 m/s before the testing. A vertical squat jump test (maximal jump height among three attempts), as the baseline, was used to identify when the participant was being fatigued (*21*). Following the jumps, the participant walked on the treadmill at 1.18 m/s and 0° of incline for 5 min, which was repeated after the participant was being fatigued. During the motor fatiguing protocol, the inclination angle of the treadmill was increased by 2.5° every 5 min until the participant had a Borg RPE > 17/20 and reached ~85% of their maximum age-predicted heart rate or due to voluntary exhaustion, where they expressed to pause the protocol due to fatigue. After the conditions were met, we paused the motor fatiguing protocol and asked the participant to perform three vertical squat jumps. If participant’s vertical jump height was reduced by 20%, the motor fatiguing protocol was terminated. Otherwise, the motor fatiguing protocol resumed from the last incline setting. We name this the fatigue modeling dataset. In addition, we included a vision calibration pipeline: We recorded the YSuit data and a mono-camera video (Apple iPhone 13 Pro) while the wearer was implementing a 4-min radio calisthenics. We name this the vision calibration dataset.

### Data preprocessing

For the accuracy study, pattern recognition study, and vision calibration pipeline, we preprocessed the YSuit data by linearly interpolating sensor values to 100 Hz (60 Hz for vision calibration pipeline) using the function of *interp* in the Python Numpy (https://numpy.org/doc/stable/reference/generated/numpy.interp.html) package. We used a visual 3D software (C-Motion Inc., Germantown, MD, USA) to extract the joint angles from the optical motion capture system (100 Hz). This process includes labeling markers, data filtering with a fourth order Butterworth low-pass filter (cutoff frequency of 10 Hz) to eliminate marker vibration caused by inertia effects and joint angle extraction. We synchronized the YSuit data with the O-MoCap informed joint angles by visually matching the peaks at the beginning and the end of data collection procedures, where we instructed the wearer to squat five times at the beginning and the end of each data collection procedure. For the pattern recognition study, we assigned respective motion classes to each frame of motions (100 Hz). For the vision calibration pipeline, we extracted video frames at 60 Hz and visually synchronized them with linearly interpolated YSuit data.

### Data processing for accuracy study

The single joint motion dataset in Fig. 3 (A and B) has 38,700 samples (387 s) in total; the multijoint motion dataset in Figs. 3C and 4C has 20,200 samples (202 s) in total; the drift mitigation dataset in Fig. 4D has 19,850 samples (198.5 s) in total. All these datasets are randomly split into training, validation, and test subdatasets with a ratio of 3:1:1, respectively. The seven rounds of 1-min single-joint movement dataset in Fig. 4 (A and B) has 40,000 samples (400 s) in total, with the first 10,200 samples (two rounds) as training data and the following 26,900 samples (five rounds) as test data. We used a standard MLP with five fully connected hidden layers, each comprising 100 rectified linear units. The training involved mean-squared error loss, Adam optimizer (learning rate: 1 × 10^−3^; epsilon: 10^−4^), and batch size of 64 in 10,000 iterations. For the qualitative sensor drift analysis in Fig. 4Da and figs. S5 and S6, we smooth out the sensor responses due to movements using the function of *savgol_filter* in the Python Scipy (https://docs.scipy.org/doc/scipy/reference/generated/scipy.signal.savgol_filter.html) package twice with a time window of 18,000 and mode of nearest.

### Data processing for pattern recognition study

The upper-body pick-and-place dataset of 1.0× speed (90 BPM) in Fig. 5A has 24,600 samples; the lower-body walking dataset of 1.0× speed (1.18 m/s) with regular step width in Fig. 5B has 17,750 samples. All these datasets are randomly split into training, validation, and test subdatasets with a ratio of 3:1:1, respectively. The upper-body pick-and-place dataset has 15 classes of upper position at 0.6×, 0.8×, 1.0×, 1.2×, and 1.4× speed (1.0× = 90 BPM) with (5740, 5775, 5795, 5990, and 5950) samples, center position with (5400, 6225, 5525, 5820, and 5850) samples, and lower position with (5700, 5350, 6120, 5890, and 5680) samples. The lower-body walking dataset has 15 classes of regular step width at 0.6×, 0.8×, 1.0×, and 1.2× speed (1.0× = 1.18 m/s); narrow-, narrow, regular, wide, and sway step width at 1.0× speed; slopes of −6°, −3°, 0°, 3°, 6°, and 9° at 1.0× speed, and a regular step width. Each class has 18,200 samples. All the samples are split into training, validation, and test datasets with a ratio of 3:1:1 in the time sequential order. We used a LSTM recurrent neural network with three layers, a hidden size of 50, one fully connected layer, and a Softmax operator. The training involved cross-entropy loss, Adam optimizer (learning rate: 1 × 10^−3^; epsilon: 10^−4^), one look-back step, and batch size of 64 in 1000 iterations. We used the *fft* function in the Python Scipy (https://docs.scipy.org/doc/scipy/tutorial/fft.html) package to implement frequency analysis in Fig. 5, Ac and Bb, and figs. S7B and S10B.

### Data processing for application study

YSuit raw sensor readings are used in Fig. 6 for qualitative evaluation. We used the same *fft* function to implement frequency analysis for Fig. 7E and fig. S11Ab. For the large language models study in Fig. 8, we used the trained machine learning models from the accuracy studies in Fig. 3 to predict all the body joint angles on the test datasets, rendered the digital human motions in video frames, and let the GPT-4V model interpret the motions in words as shown in Fig. 8.

### Ethics statement

The participant gave written informed consent to participate in the study. The study complied with the Declaration of Helsinki and was approved by the Institutional Review Board of the University of Massachusetts Lowell (protocol #20-057).

### AI use

A GPT-4V-based pipeline is developed to interpret human motions into verbal descriptions, as shown in Fig. 8 and explained in the Large language model (GPT-4V) section.

## References

[1] C. Tang, W. Yi, E. Occhipinti, Y. Dai, S. Gao, L. G. Occhipinti, A roadmap for the development of human body digital twins. Nat. Rev. Electr. Eng. 1, 199–207 (2024).

[2] S. Xu, J. Kim, J. R. Walter, R. Ghaffari, J. A. Rogers, Translational gaps and opportunities for medical wearables in digital health. Sci. Transl. Med. 14, eabn6036 (2022). 36223451 10.1126/scitranslmed.abn6036 PMC10193448

[3] H. C. Ates, P. Q. Nguyen, L. Gonzalez-Macia, E. Morales-Narváez, F. Güder, J. J. Collins, C. Dincer, End-to-end design of wearable sensors. Nat. Rev. Mater. 7, 887–907 (2022). 35910814 10.1038/s41578-022-00460-x PMC9306444

[4] J. Bai, L. Shen, H. Sun, B. Shen, *Physiological Informatics: Collection and Analyses of Data from Wearable Sensors and Smartphone for Healthcare* (Springer, 2017), pp. 17–37. 10.1007/978-981-10-6041-0_2 29058214

[5] Z. Zhang, J. Yang, H. Wang, C. Wang, Y. Gu, Y. Xu, S. Lee, T. Yokota, H. Haick, T. Someya, Y. Wang, A 10-micrometer-thick nanomesh-reinforced gas-permeable hydrogel skin sensor for long-term electrophysiological monitoring. Sci. Adv. 10, eadj5389 (2024). 38198560 10.1126/sciadv.adj5389 PMC10781413

[6] E. A. Tarim, B. Erimez, M. Degirmenci, H. Cumhur Tekin, A wearable device integrated with deep learning-based algorithms for the analysis of breath patterns. Adv. Intell. Syst. 5, 2300174 (2023).

[7] H. Lee, E. Kim, Y. Lee, H. Kim, J. Lee, M. Kim, H.-J. Yoo, S. Yoo, Toward all-day wearable health monitoring: An ultralow-power, reflective organic pulse oximetry sensing patch. Sci. Adv. 4, eaas9530 (2018). 30430132 10.1126/sciadv.aas9530 PMC6226280

[8] Y. Song, J. Min, Y. Yu, H. Wang, Y. Yang, H. Zhang, W. Gao, Wireless battery-free wearable sweat sensor powered by human motion. Sci. Adv. 6, eaay9842 (2020). 32998888 10.1126/sciadv.aay9842 PMC7527225

[9] M. Kang, H. Jeong, S.-W. Park, J. Hong, H. Lee, Y. Chae, S. Yang, J.-H. Ahn, Wireless graphene-based thermal patch for obtaining temperature distribution and performing thermography. Sci. Adv. 8, eabm6693 (2022). 35417247 10.1126/sciadv.abm6693 PMC9007510

[10] S. Gedam, S. Paul, A review on mental stress detection using wearable sensors and machine learning techniques. IEEE Access 9, 84045–84066 (2021).

[11] F.-T. Sun, C. Kuo, H.-T. Cheng, S. Buthpitiya, P. Collins, M. Griss, Activity-aware mental stress detection using physiological sensors, in *Mobile Computing, Applications, and Services*, M. Gris, G. Yang, Eds. (Springer, 2012), pp. 282–301.

[12] J. Pärkkä, M. Ermes, M. van Gils, Automatic feature selection and classification of physical and mental load using data from wearable sensors, in *Proceedings of the 10th IEEE International Conference on Information Technology and Applications in Biomedicine* (IEEE, 2010), pp. 1–5.

[13] G. M. Harari, S. D. Gosling, Understanding behaviours in context using mobile sensing. Nat. Rev. Psychol. 2, 767–779 (2023).

[14] F. Serpush, M. B. Menhaj, B. Masoumi, B. Karasfi, Wearable sensor-based human activity recognition in the smart healthcare system. Comput. Intell. Neurosci. 2022, 1391906 (2022). 35251142 10.1155/2022/1391906 PMC8894054

[15] S. Ancona, F. D. Faraci, E. Khatab, L. Fiorillo, O. Gnarra, T. Nef, C. L. A. Bassetti, P. Bargiotas, Wearables in the home-based assessment of abnormal movements in parkinson’s disease: A systematic review of the literature. J. Neurol. 269, 100–110 (2022). 33409603 10.1007/s00415-020-10350-3

[16] Z. H. Lewis, L. Pritting, A.-L. Picazo, M. JeanMarie-Tucker, The utility of wearable fitness trackers and implications for increased engagement: An exploratory, mixed methods observational study. Digit. Health 6, 2055207619900059 (2020). 31976079 10.1177/2055207619900059 PMC6958644

[17] A. Zadeh, D. Taylor, M. Bertsos, T. Tillman, N. Nosoudi, S. Bruce, Predicting sports injuries with wearable technology and data analysis. Inf. Syst. Front. 23, 1023–1037 (2021).

[18] M. Rana, V. Mittal, Wearable sensors for real-time kinematics analysis in sports: A review. IEEE Sens. J. 21, 1187–1207 (2021).

[19] H. Kim, Y.-T. Kwon, H.-R. Lim, J.-H. Kim, Y.-S. Kim, W.-H. Yeo, Recent advances in wearable sensors and integrated functional devices for virtual and augmented reality applications. Adv. Funct. Mater. 31, 2005692 (2021).

[20] K. Ogata, Y. Matsumoto, Estimating movements of human body for the shirt-type wearable device mounted on the strain sensors based on convolutional neural networks, in *Proceedings of the 41st Annual International Conference of the IEEE Engineering in Medicine and Biology Society (EMBC)* (IEEE, 2019), pp. 5871–5876. 10.1109/EMBC.2019.8856722 31947186

[21] P.-C. Kao, C. Lomasney, Y. Gu, J. P. Clark, H. A. Yanco, Effects of induced motor fatigue on walking mechanics and energetics. J. Biomech. 156, 111688 (2023). 37339542 10.1016/j.jbiomech.2023.111688

[22] C. Siviy, L. M. Baker, B. T. Quinlivan, F. Porciuncula, K. Swaminathan, L. N. Awad, C. J. Walsh, Opportunities and challenges in the development of exoskeletons for locomotor assistance. Nat. Biomed. Eng. 7, 456–472 (2023). 36550303 10.1038/s41551-022-00984-1 PMC11536595

[23] J. L. Pons, Witnessing a wearables transition. Science 365, 636–637 (2019). 31416946 10.1126/science.aaw9407

[24] G. Maugeri, P. Castrogiovanni, G. Battaglia, R. Pippi, V. D’Agata, A. Palma, M. Di Rosa, G. Musumeci, The impact of physical activity on psychological health during covid-19 pandemic in italy. Heliyon 6, e04315 (2020). 32613133 10.1016/j.heliyon.2020.e04315 PMC7311901

[25] I. Roupa, M. R. da Silva, F. Marques, S. B. Gonçalves, P. Flores, M. T. da Silva, On the modeling of biomechanical systems for human movement analysis: A narrative review. Arch. Comput. Methods Eng. 29, 4915–4958 (2022).

[26] M. P. Silvernagel, A. S. Ling, P. Nuyujukian, Brain Interfacing Laboratory, A markerless platform for ambulatory systems neuroscience. Sci. Robot. 6, eabj7045 (2021). 34516749 10.1126/scirobotics.abj7045

[27] M. Kennedy III, The role of collaborative robotics in assistive and rehabilitation applications. Sci. Robot. 8, eadk6743 (2023). 37878691 10.1126/scirobotics.adk6743

[28] V. Ricotti, B. Kadirvelu, V. Selby, R. Festenstein, E. Mercuri, T. Voit, A. A. Faisal, Wearable full-body motion tracking of activities of daily living predicts disease trajectory in duchenne muscular dystrophy. Nat. Med. 29, 95–103 (2023). 36658421 10.1038/s41591-022-02045-1 PMC9873561

[29] D. Aarsland, L. Batzu, G. M. Halliday, G. J. Geurtsen, C. Ballard, K. Ray Chaudhuri, D. Weintraub, Parkinson disease-associated cognitive impairment. Nat. Rev. Dis. Primers 7, 47 (2021). 34210995 10.1038/s41572-021-00280-3

[30] N. Kuzik, P.-J. Naylor, J. C. Spence, V. Carson, Movement behaviours and physical, cognitive, and social-emotional development in preschool-aged children: Cross-sectional associations using compositional analyses. PLOS ONE 15, e0237945 (2020). 32810172 10.1371/journal.pone.0237945 PMC7433874

[31] F. Roggio, S. Ravalli, G. Maugeri, A. Bianco, A. Palma, M. Di Rosa, G. Musumeci, Technological advancements in the analysis of human motion and posture management through digital devices. World J. Orthop. 12, 467–484 (2021). 34354935 10.5312/wjo.v12.i7.467 PMC8316840

[32] Z. Fan, O. Taheri, D. Tzionas, M. Kocabas, M. Kaufmann, M. J. Black, O. Hilliges, ARCTIC: A dataset for dexterous bimanual hand-object manipulation, in *Proceedings of the IEEE Conference on Computer Vision and Pattern Recognition (CVPR)* (IEEE, 2023).

[33] B. R. Hindle, J. W. L. Keogh, A. V. Lorimer, Inertial-based human motion capture: A technical summary of current processing methodologies for spatiotemporal and kinematic measures. Appl. Bionics Biomech. 2021, 6628320 (2021). 33859720 10.1155/2021/6628320 PMC8024877

[34] M. Kaufmann, Y. Zhao, C. Tang, L. Tao, C. Twigg, J. Song, R. Wang,O. Hilliges, Em pose: 3d human pose estimation from sparse electromagnetic trackers, in *Proceedings of the IEEE/CVF International Conference on Computer Vision (ICCV)* (IEEE, 2021), pp. 11490–11500.

[35] E. van der Kruk, M. M. Reijne, Accuracy of human motion capture systems for sport applications; state-of-the-art review. Eur. J. Sport Sci. 18, 806–819 (2018). 29741985 10.1080/17461391.2018.1463397

[36] T. Fukuoka, S. Irie, Y. Watanabe, T. Kutsuna, A. Abe, The relationship between spatiotemporal gait parameters and cognitive function in healthy adults: Protocol for a cross-sectional study. Pilot Feasibility Stud. 8, 154 (2022). 35879785 10.1186/s40814-022-01122-z PMC9310397

[37] T. von Marcard, B. Rosenhahn, M. J. Black, G. Pons-Moll, Sparse inertial poser: Automatic 3d human pose estimation from sparse imus. Comput. Graph. Forum 36, 349–360 (2017).

[38] D. Kim, J. Kwon, S. Han, Y.-L. Park, S. Jo, Deep full-body motion network for a soft wearable motion sensing suit. IEEE/ASME Trans. Mechatron. 24, 56–66 (2019).

[39] O. Glauser, S. Wu, D. Panozzo, O. Hilliges, O. Sorkine-Hornung, Interactive hand pose estimation using a stretch-sensing soft glove. ACM Trans. Graph. 38, 1–15 (2019).

[40] A. Tashakori, Z. Jiang, A. Servati, S. Soltanian, H. Narayana, K. Le, C. Nakayama, C.-l. Yang, Z. J. Wang, J. J. Eng, P. Servati, Capturing complex hand movements and object interactions using machine learning-powered stretchable smart textile gloves. Nat. Mach. Intell. 6, 106–118 (2024).

[41] R. Lin, H.-J. Kim, S. Achavananthadith, Z. Xiong, J. K. W. Lee, Y. L. Kong, J. S. Ho, Digitally-embroidered liquid metal electronic textiles for wearable wireless systems. Nat. Commun. 13, 2190 (2022). 35449159 10.1038/s41467-022-29859-4 PMC9023486

[42] Y. Zhao, K. Zhao, R. Qian, Z. Yu, C. Ye, Interfacial engineering of liquid metal nanoparticles for the fabrication of conductive hydrogels: A review. Chem. Eng. J. 486, 150197 (2024).

[43] N. Zhang, G. Xiong, Z. Liu, Toxicity of metal-based nanoparticles: Challenges in the nano era. Front. Bioeng. Biotechnol. 10, 1001572 (2022). 36619393 10.3389/fbioe.2022.1001572 PMC9822575

[44] T. Nishikawa, H. Yamane, N. Matsuhisa, N. Miki, Stretchable strain sensor with small but sufficient adhesion to skin. Sensors 23, 1774 (2023). 36850371 10.3390/s23041774 PMC9967902

[45] B. Zhang, J. Li, J. Zhou, L. Chow, G. Zhao, Y. Huang, Z. Ma, Q. Zhang, Y. Yang, C. K. Yiu, J. Li, F. Chun, X. Huang, Y. Gao, P. Wu, S. Jia, H. Li, D. Li, Y. Liu, K. Yao, R. Shi, Z. Chen, B. L. Khoo, W. Yang, F. Wang, Z. Zheng, Z. Wang, X. Yu, A three-dimensional liquid diode for soft, integrated permeable electronics. Nature 628, 84–92 (2024). 38538792 10.1038/s41586-024-07161-1

[46] Y. Luo, Y. Li, P. Sharma, W. Shou, K. Wu, M. Foshey, B. Li, T. Palacios, A. Torralba, W. Matusik, Learning human–environment interactions using conformal tactile textiles. Nat. Electron. 4, 193–201 (2021).

[47] H. Lee, H. Sun, H. Park, G. Serhat, B. Javot, G. Martius, K. J. Kuchenbecker, Predicting the force map of an ert-based tactile sensor using simulation and deep networks. IEEE Trans Autom. Sci. Eng. 20, 425–439 (2023).

[48] L. Sanchez-Botero, A. Agrawala, R. Kramer-Bottiglio, Stretchable, breathable, and washable fabric sensor for human motion monitoring. Adv. Mater. Technol. 8, 2300378 (2023).

[49] G. Pavlakos, V. Choutas, N. Ghorbani, T. Bolkart, A. A. A. Osman, D. Tzionas, M. J. Black, Expressive body capture: 3D hands, face, and body from a single image, in *Proceedings of the IEEE Conference on Computer Vision and Pattern Recognition (CVPR)* (IEEE, 2019), pp. 10975–10985.

[50] N. Mahmood, N. Ghorbani, N. F. Troje, G. Pons-Moll, M. J. Black, AMASS: Archive of motion capture as surface shapes, in *Proceedings of the IEEE International Conference on Computer Vision* (IEEE, 2019), pp. 5442–5451.

[51] W. contributors, “Radio calisthenics” (2024); https://en.wikipedia.org/wiki/Radio_calisthenics.

[52] N. A. Coull, A. M. West, S. G. Hodder, P. Wheeler, G. Havenith, Body mapping of regional sweat distribution in young and older males. Eur. J. Appl. Physiol. 121, 109–125 (2021). 32990756 10.1007/s00421-020-04503-5 PMC7815578

[53] S. Hochreiter, J. Schmidhuber, Long Short-Term Memory. Neural Comput. 9, 1735–1780 (1997). 9377276 10.1162/neco.1997.9.8.1735

