# Supplementary material for: Textile suit for anywhere full-body motion capture
Source: Sci Adv. 2026 Mar 4;12(10):eaea2646. doi: 10.1126/sciadv.aea2646 (PMC12959391; doi:10.1126/sciadv.aea2646)
Supplement: Supplementary file 1 — Supplementary Text Figs. S1 to S11 Legend for movie S1 [file sciadv.aea2646_sm.pdf]

Science Advances
AAAS

Supplementary Materials for

Textile suit for anywhere full-body motion capture

Huanbo Sun *et al.*

Corresponding author: Huanbo Sun, huanbo.sun@pku.edu.cn;
Rebecca Kramer-Bottiglio, rebecca.kramer@yale.edu

Sci. Adv. **12**, eaea2646 (2026)
DOI: 10.1126/sciadv.aea2646

The PDF file includes:

Supplementary Text
Figs. S1 to S11
Legend for movie S1

Other Supplementary Material for this manuscript includes the following:

Movie S1

Supplementary Text

Sensor Unit Characterization The green "Signal" layer of our sensor (Fig. 2 A**a**) is positively charged, while the red "Grounding" layer is negatively charged. The "Signal" layer carries twice the charge per unit area compared to the "Grounding" layer. The capacitance is determined by the relation:

$$C = Q/V = I \times t/V, \quad (1)$$

where Q is the charge, I is the programmable charge current, t is the charge duration, and V is the measured voltage across the layers. Once the electrode is charged, the peak voltage V is measured using an internal 10-bit ADC. This voltage (represented as ADC counts) is inversely proportional to the capacitance C of the sensing channel. The data acquisition (DAQ) system measures the voltage difference between the Signal and Grounding layers. The capacitance of our textile sensor is proportional to the sensing area (length $\times$ width), as shown in Fig. S 1 A**a**, where we maintained a constant sensor length of 160 mm while varying the sensor width. The capacitance measures approximately 100 pF when the sensor dimensions are 160 $\times$ 10 mm. We used the Adafruit MPR121 as the data acquisition (DAQ) system to record the sensor capacitance, configuring the MPR121 registers with MPR121_CONFIG1 set to 0$\times$10 (I: 16 uA charge current) and MPR121_CONFIG2 set to 0$\times$20 (t: 0.5 uS encoding). As shown in Fig. S 1 A**b**, the DAQ system offers a resolution of 10 bits (1024), yet its readings are not linearly proportional to the capacitance (represented by the red line). To derive the sensitivity curve, we calculated the first derivative of the red curve. The sensitivity peaks at 57 pF and diminishes gradually on either side. Capacitance values below this threshold lead to ADC saturation and are therefore unsuitable. Values above 57 pF remain within the acceptable range. To ensure a balanced response across the 0–1024 ADC range, we selected a capacitance of approximately 100 pF at no strain and 150 pF at 50% strain, centering the signal around 512—midway in the

ADC range. During the initial 50% strain (extending the sensor by 80 mm), both the capacitance and pulling force increase linearly with the sensor width, as demonstrated in Fig. S 1 A**c**. To balance the DAQ's measurement range, sensor sensitivity, and the required actuation force, we decided that maintaining a sensor form factor 160×10 mm is ideal with a base capacitance of ~ 100 pF. Adding fabric trace to the sensor increases total capacitance, and both trace width and length contribute linearly to the increase, as shown in Fig. S 1 A**d**.

Our wearable suit is designed for multiple uses, necessitating careful consideration of its long-term application. We observed slight sensor drift (7%) over one week of inactivity, as shown in Fig. S 1 B**b**, where the behavior during the second 100 stretch cycles reflects the sensor's response after one week compared to the first 100 cycles. However, as shown in Fig. S 1 C**b**, the sensor's behavior can recover after washing and drying. This recovery is due to the viscoelastic nature of the nylon and spandex fibers: cyclic stretching causes microscopic deformations in the fiber chains, raising the base capacitance, while heat and mechanical agitation from washing allow the fibers to relax back to their original, unstretched state. As shown in Fig. 2 (A**c**), Fig. S 1 B**c**, and Fig. S 1 C**c**, the sensor capacitance increases monotonically with applied strain, with a slightly higher rate of increase under greater strain, indicating a non-linear relationship. As established by Eqn. S(1), the electric field distribution V is inversely proportional to the capacitance C, and our measurements (Fig. S1 Bc, Cc, Dc) show that applied strain increases the capacitance. Consequently, the electric field magnitude decreases with increasing strain, which experimentally manifests as a reduction in the measured voltage. This provides a clear mechanistic link between strain, capacitance, and voltage readout, clarifying the effects of stretching on sensor performance.

In addition, the observed mechanical hysteresis manifests as a divergence between the loading and unloading curves, where both the capacitance and force values are consistently higher during the loading phase for a given strain level (Fig. S 1 B**d** and Fig. S 1 C**d**). Furthermore, our

results demonstrate that the magnitude of this hysteresis decreases with repeated cyclic loading, indicating a mechanical conditioning or “training” effect in the material.

Moreover, the laser-cut fabric traces forms melted edges, which can contribute to mechanical instability. The pre-stretching process helps break and stabilize these melted edge structures. To address this, we apply an automated pre-conditioning step of 100 cycles at 50% strain before assembling the sensor units and fabric traces onto the garment. This minimizes drift caused by edge effects and improves long-term measurement stability.

Sensing Network The customization pipeline optimizes the placement position for each joint orientation direction, determining the ideal location for each textile sensor unit corresponding to each direction. In addition to sensor noise within each unit, the traces connecting the sensors to the DAQ system also induce capacitance changes (Fig. S 1 E), along with parasitic capacitance between nearby sensors and traces. Furthermore, human body skin stretch and bending occur across multiple planes, activating local textile sensors. Consequently, each sensor unit is influenced by movements in other areas of the body. These combined factors impact the sensor readings specific to individual joint orientations. Therefore, we propose utilizing all 38 textile sensor units to jointly predict individual joint rotation directions. We used the k-Nearest Neighbors (kNN) algorithm to greedily select the most impactful sensors for predicting each joint angle, ranking their contribution to improving prediction accuracy. As depicted in Fig. S 3 A, involving more sensors in the prediction leads to smaller errors; even three sensors can significantly enhance accuracy. All angle errors for every joint are smaller than 1.5°. Moreover, analysing each sensor’s contribution to specific joints allows us to identify correlations between joint movements. This process is facilitated by the sensing network formed by multiple sensor units.

Theorectial Accuracy Reference Each sensor provides a measurement: θ_n. Each sensor's measurement error is independent, unbiased (meaning the error has a mean of zero), and normally distributed with a standard deviation of σ_S. The sensor measurement can be written as:

$$\theta_n = \theta_{true} + \epsilon_n, \text{ where } \epsilon_n \sim N(0, \sigma_S^2).$$

As shown in Fig. 2 A**d** and Fig. S 1 D**c**, the initial sensor noise, characterized by a standard deviation of **1.8** degrees, was measured across the joint's 0-90° range of motion. For each joint, we placed three sensors to measure the angle change. Thus, the fused accuracy is:

$$\theta_{fused} = (\theta_1 + \theta_2 + \theta_3)/3 + \epsilon_{fused}, \text{ where } \epsilon_{fused} \sim N(0, \sigma_S^2/3).$$

Consequently, the theoretical lower bound for the standard deviation of the joint angle measurement, achieved by optimally fusing the three sensors, is $1.8/sqrt(3) =$ **1.04** degrees. This demonstrates that integrating additional sensors improves measurement accuracy, as the uncertainty scales inversely with the square root of the number of sensors. The value of 1.04 degrees closely matches the empirical results from our single-joint motion evaluation (Fig. 3 A and B), where the observed errors were approximately 1.1 degrees or slightly higher. This theoretical analysis provides a crucial baseline for evaluating our data processing methods.

Calibration Model Comparison For the single joint motion calibration depicted in Fig. 3A, we compared the performance of MLP with a kNN (two neighbours) method. kNN operates by storing all training data in memory, akin to a lookup table, and computes the averaged value of the closest samples in the stored data for the new data sample. As shown in Fig. S 3 B**a** and B**b**, kNN demonstrates slightly superior performance compared to MLP, but requires much more storage space (3.14 MB versus 236 KB). The performance of both models can be enhanced with more data points, as shown in Fig. S 3 B**c** and B**d**. However, this necessitates additional storage space for the kNN model, which is not the case for MLP. Consequently, we utilized

MLP to process the interpolated sensor data for all our accuracy studies. Further comparisons are summarized in Fig. S 4 , and all the aforementioned conclusions remain consistent: the performance of MLP improves with more data but necessitates the same storage space.

Drift Analysis When we intensify our pace during activities involving increased joint movement and persist for an extended duration, additional physiological responses may emerge, including sweating. Our preliminary study on the sensor response for simplified sweat analysis indicates that it is influenced by moisture. For the qualitative sensor drift analysis in Fig. 4 D**a**, Fig. S 5 , and Fig. S 6 , we mitigate the effects of movement on sensor responses using the *savgol_filter* function, applying it twice with a time window of 18,000 and a mode of nearest. All sensor readings for each joint over a 16-minute period are depicted in Fig. S 5 . Each sub-figure comprises a left plot showing raw sensor readings with fitted smoothed solid curves, and a right plot illustrating drift-filtered sensor readings. This represents a qualitative separation of the drift effect from the movements. Across various joints, distinct drift trends emerge over time: sensors near the upper arms, shoulders, thighs, and knees exhibit more significant drift than others, with the lower back following; additionally, the right side displays more pronounced drift compared to the left. Figure S 6 illustrates the temporal evolution of drift across space. These qualitative findings align with the quantitative analysis presented in (*52*), indicating the suit's potential for monitoring the spatial distribution of sweat. Further exploration of this capability is a direction for future investigation.

Currently, the system does not support real-time, frame-by-frame pose reconstruction under heavy sweating. For non-intensive motion capture without significant perspiration, accuracy remains high, with an average error below 2 degrees. In heavy sweat conditions, motion signals cannot be fully separated from sweat-induced drift with the current hardware, and even adding a sweat sensor cannot achieve real-time correction due to the absence of detailed spatial mapping

of localized sweat distribution.

For offline analysis, however, drift can be effectively mitigated. As shown in Fig. 4 D**a**, Fig. S 5 , and Fig. S 6 , we apply low-pass filtering and baseline drift correction to isolate slow sweat-related variations while preserving motion signals. Furthermore, FFT-based analysis (Fig. 5 , Fig. 7 E, Fig. S 7 , Fig. S 10 , Fig. S 11) enables frequency-domain examination of limb and joint activity. Because sweat-induced drift occurs only at very low frequencies, it is effectively removed, allowing focus on motion-relevant signals

Note that our operational definition of "non-intensive motion" is kinematic rather than categorical. The $< 2°$ accuracy claim refers specifically to motion regimes with ~ 3.9–$5.9°/10\,\mathrm{ms}$ single-joint or $\leq 15.9°/10\,\mathrm{ms}$ multi-joint angular change rates, corresponding to periods before sweat-induced drift develops. Because sweating evolves gradually and depends on factors such as activity duration, metabolism, and emotional state, it does not admit a single physiological cutoff for heavy versus non-intensive conditions.

Upper Body Pick & Place Analysis To evaluate the joint accuracy for upper body pick and place analysis, we conducted additional ablation studies, as illustrated in Fig. S 7 A. For the speed set at $1.0\times$ (90 BPM), the wearer performed repetitive movements for the upper, middle, and lower heights individually for one minute each, followed by a one-minute session incorporating a mixture of all movement heights at varying combinations. We systematically ablated the dataset to assess the generalizability of these movements to alternative settings. When utilizing the entire movement dataset, accuracy was within angle errors below 2 degrees (except for elbows). However, when attempting to predict mixture movements using solely upper, middle, and lower height data, accuracy notably declined, with angle errors tripling. This decrease in performance can likely be attributed to the transitioning between different height levels, a scenario not adequately represented in the isolated repetitive movements dataset. Conversely,

when attempting to predict individual upper, middle, and lower height movements using mixture movement data, accuracy worsened further, with angle errors quadrupling. This decline can be attributed to both the limited sample size within the one-minute mixture movement dataset and the discrepancy in motion distributions among the different datasets. The key takeaway for improving the accuracy is to collect copious good data, covering a wide range of poses and transition speeds between movements.

In addition to predicting joint angles frame by frame, we also analyzed the raw YSuit data in the frequency domain to provide an alternative perspective on human movements. We used a fast Fourier transformation to extract the amplitude and frequency of each sensor, which allowed us to analyze the local joint movement amplitude and speed, as shown in Fig. S 7 B. For movements at different heights at the same speed (e.g., $0.6\times$), the wearer engaged different joint combinations: shoulders more for upper height, elbows more for middle height, and both shoulders and elbows (with a slight emphasis on shoulders) for lower height, as shown in Fig. S 7 B**a**. This trend remained consistent across all speeds, and a similar pattern was observed in both the left and right limbs. It is noteworthy that at different speeds, there was a reallocation of joint movements by the wearer. Figure S 7 B**b** displays the sensor responses at different speeds, which are clearly distinguishable.

Moreover, understanding movement patterns over certain periods enables us to discern human activities and enhance performance. In our study, we introduced the Long Short-Term Memory (LSTM) network structure in the main text to capture the time dependence in motion data. As part of an ablation study, we also used a k-Nearest Neighbors (kNN) model and a Multi-Layer Perceptron (MLP) model for the pattern recognition task; these comparisons are presented in Fig. S 9 A. Overall, the LSTM model outperforms both the MLP and kNN models, with the MLP model performing better than the kNN model. This result can primarily be attributed to two factors: (1) similarity between poses during motion across different movement classes, and (2)

nonlinearity in the data samples, where kNN averages over the closest two data samples while MLP finds non-linear interpolation between data samples. The LSTM uses a “lookback step" parameter to establish time dependence and control the length of historical data considered to predict the current status. We further investigated the length of the lookback step, as shown in Fig. S 9 B. With only one lookback step, the accuracy converges to a very high level during the training procedure, with small fluctuations over time, owing to the similarity in short-time movement patterns. With increased length, more time-steps are taken into account, tending to improve accuracy with a slower convergence speed during the training procedure. However, when the length is comparatively long, the accuracy drops because finding a long time-sequence match between test data and training data is non-trivial. This trade-off of the lookback step needs to be considered. For our main results, we used a lookback step length of one.

Lower Body Walking Analysis The lower-body walking analysis follows a similar approach to the upper-body analysis. We compared two standard dataset splitting strategies for joint angle prediction: (1) random splitting over time sequence into training, validation, and test datasets with a ratio of 3:1:1, and (2) splitting along the time sequence with the same ratio, as shown in Fig. S 10 A**b**. In the frequency analysis, we conducted additional studies on speed, step width, and slope, as shown in Fig. S 10 B. During the speed test, the wearer increased their lower limb iteration frequency to catch up with the increased walking speed. It is worth noting that the wearer increased their lower limb bending angle to accommodate the speed change from 0.6$\times$ to 0.8$\times$, as shown in Fig. S 10 B**a**. In the step width test, the wearer had higher frequencies for narrow-, narrow, and wide step width compared to regular, because narrow- and narrow have smaller step lengths due to limited limb movements, while wide has smaller step lengths in the heading direction. For the sway, the wearer had larger bending angles and smaller frequencies. It is also interesting that the wearer’s left and right lower limb movements show asymmetry, as

shown in Fig. S 10 B**b**. In the slope test, the wearer tends to use smaller gaits and faster speed for declined slops, while the wearer tends to slow down the speed but increase thigh angles and reduce knee angles for inclined slops, as shown in Fig. S 10 B**c**.

Fatigue Modelling Analysis For the fatigue modelling analysis, we used a fast Fourier transformation (FFT) to extract the gait frequency information during the fatigue procedure. There were a total of 16 walking trials, and we applied FFT to each trial. We then used the Gaussian curve_fit function from Python Scipy to extract the mean frequencies, as illustrated in Fig. S 11 A**a**. As shown in Fig. S 11 A**b**, left pelvis (LP), left thigh (LT), left knee (LK), right knee (RK), right thigh (RT), and right pelvis (RP) data indicate that the wearer initially adapted to the procedure with decreased gait frequencies, and steadily increased them as the treadmill inclination angle increased. However, as the fatigue procedure continued, the wearer reduced the gait frequencies with less stability (Fig. 7 E, Fig. S 11 A**b**). For the overtime drift analysis in Fig. S 11 B, we used a similar approach to Fig. S 6 . The drift (Fig. S 11 B) initially occurred near the armpits and knees, then propagated to the upper and lower limbs, eventually covering the entire body, including the abdomen and back.

Large Language Models Application Interpretation Once we receive the sensor signals, our goal is to analyze them and provide explanations to users. This involves translating the signals into human poses and accurately describing these poses to understand human behavior. With the reasoning capabilities of large language models (LLMs), we can even predict intentions. However, existing models based on LLMs are not designed to interpret human poses, which are typically represented as joint angles. Instead, these models usually process textual queries or image sequences. Therefore, to leverage LLM tools like GPT-4V, we convert the YSuit signals into RGB videos. Specifically, we render RGB images using the SMPL-X pose parameters estimated from the YSuit sensor signals. This process is applied to the avatar we previously

reconstructed for the wearer, which incorporates SMPL-X shape parameters and texture colors. The pipeline is depicted in Fig. 8 . For each motion sequence, we select approximately ten key frames as input for the GPT-4-Vision-Preview API, which provides descriptions. We present examples to analyze human motion for single joints (Fig. 8 B), multiple joints (Fig. 8 C), the upper body (Fig. 8 D), and the lower body (Fig. 8 E), posing questions that require detailed descriptions or brief responses.

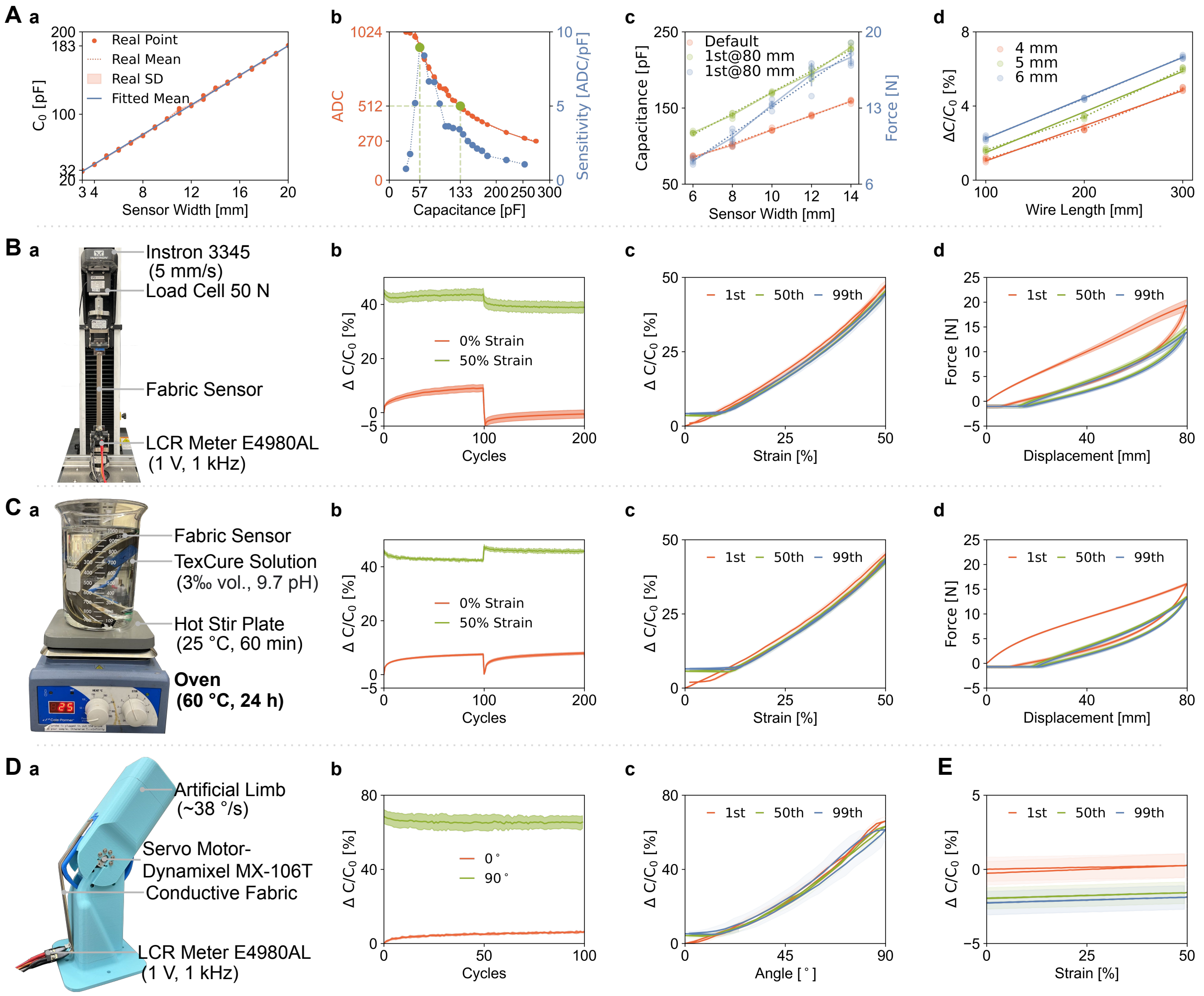

Fig. S 1. Sensor Unit Characterization. **A**, The sensor capacitance demonstrates a linear relationship with geometry width under **a** no strain and **c** 50% strain in the first cycle. However, it exhibits **b** nonlinearity in the DAQ system reading. Additionally, **d** the sensor capacitance increases linearly with both trace width and length. **B**, Sensors were subjected to a **a** stretch test with evaluations on **b** cyclic performance, **c** the relationship between displacement and capacitance change, and **d** the displacement-force relationship. **C**, Sensors underwent **a** a washing test with assessments on **b** cyclic performance, **c** the displacement-capacitance change relationship, and **d** the displacement-force relationship. **D**, Sensors were tested under **a** a bending test, with evaluations on **b** cyclic performance, and **c** the relationship between angle and capacitance change. **E**, Traces were examined under a stretch test.

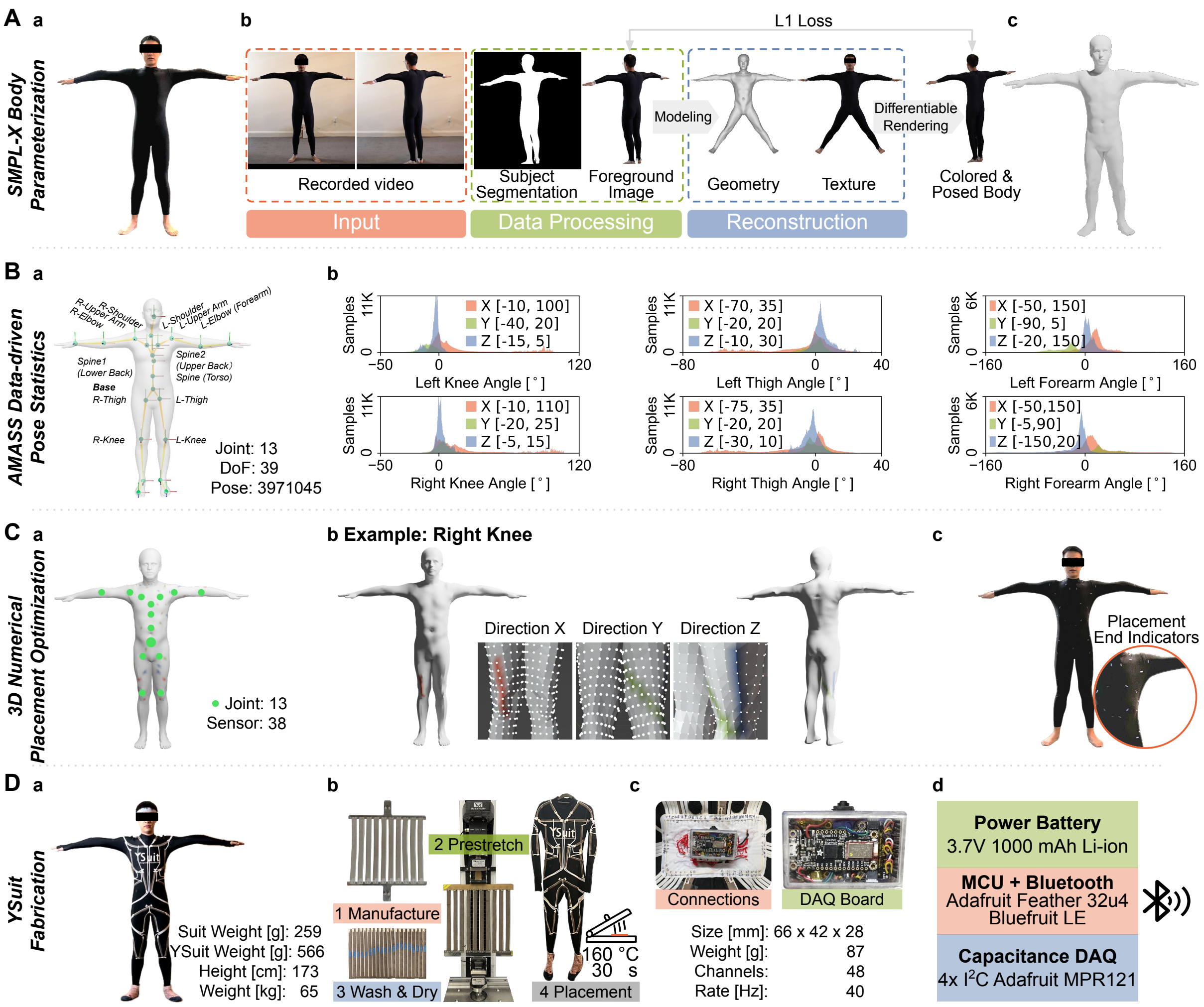

Fig. S 2. Customization Pipeline. **A**, The SMPL-X body parametrization procedure transforms **a** a real human's shape into **c** a digital 3D SMPL-X model, following the steps outlined in **b**. **B**, AMASS pose statistics analysis is conducted on **a** 13 body joints, with detailed examples provided for **b** knees, **c** thighs, and **d** forearms. **C**, Optimal sensor placement positions are numerically determined for **a** 13 body joints, with **b** the right knee as an example, and **c** markers are labelled according to the optimal placement algorithm. **D**, **a** The YSuit design and fabrication procedure includes **b** sensor preparation, **c** trace wiring to the DAQ system, and **d** setup of the DAQ system.

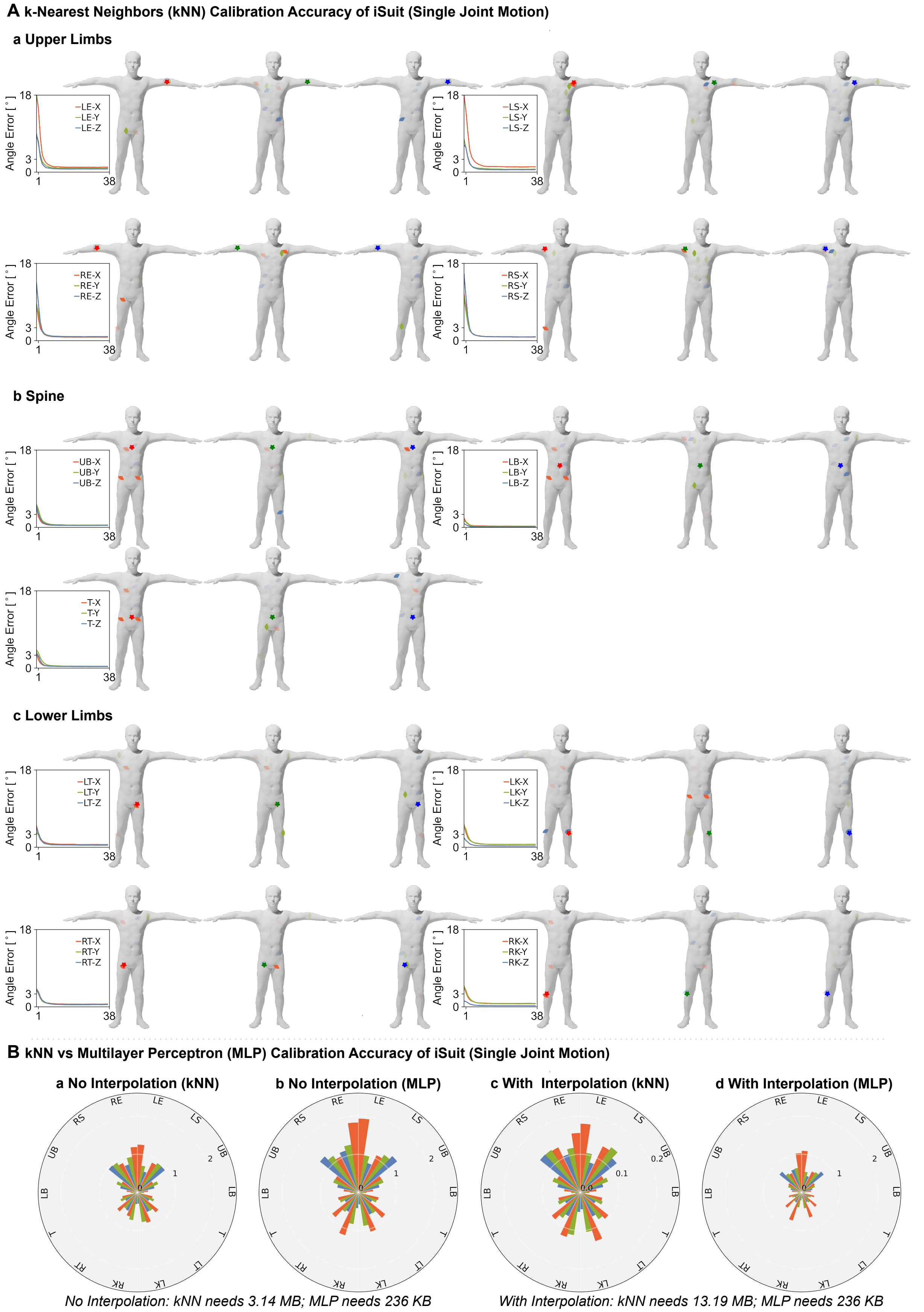

Fig. S 3. Ablation Study for YSuit Calibration. A, The sensing network formed by more sensors improves the accuracy of joint angle predictions for **a** upper limbs, **b** spine, and **c** lower limbs. The targeted joint is denoted by "★", with red, green, and blue colors representing X, Y, Z orientations, respectively. A three-part pie chart, colored in red, green, and blue, illustrates the contribution of X, Y, and Z sensors to the accuracy of the targeted joint angle prediction. Darker shades indicate a higher contribution. **B**, A comparison of kNN and MLP performance is presented for **a** the non-interpolated dataset and **b** the interpolated dataset.

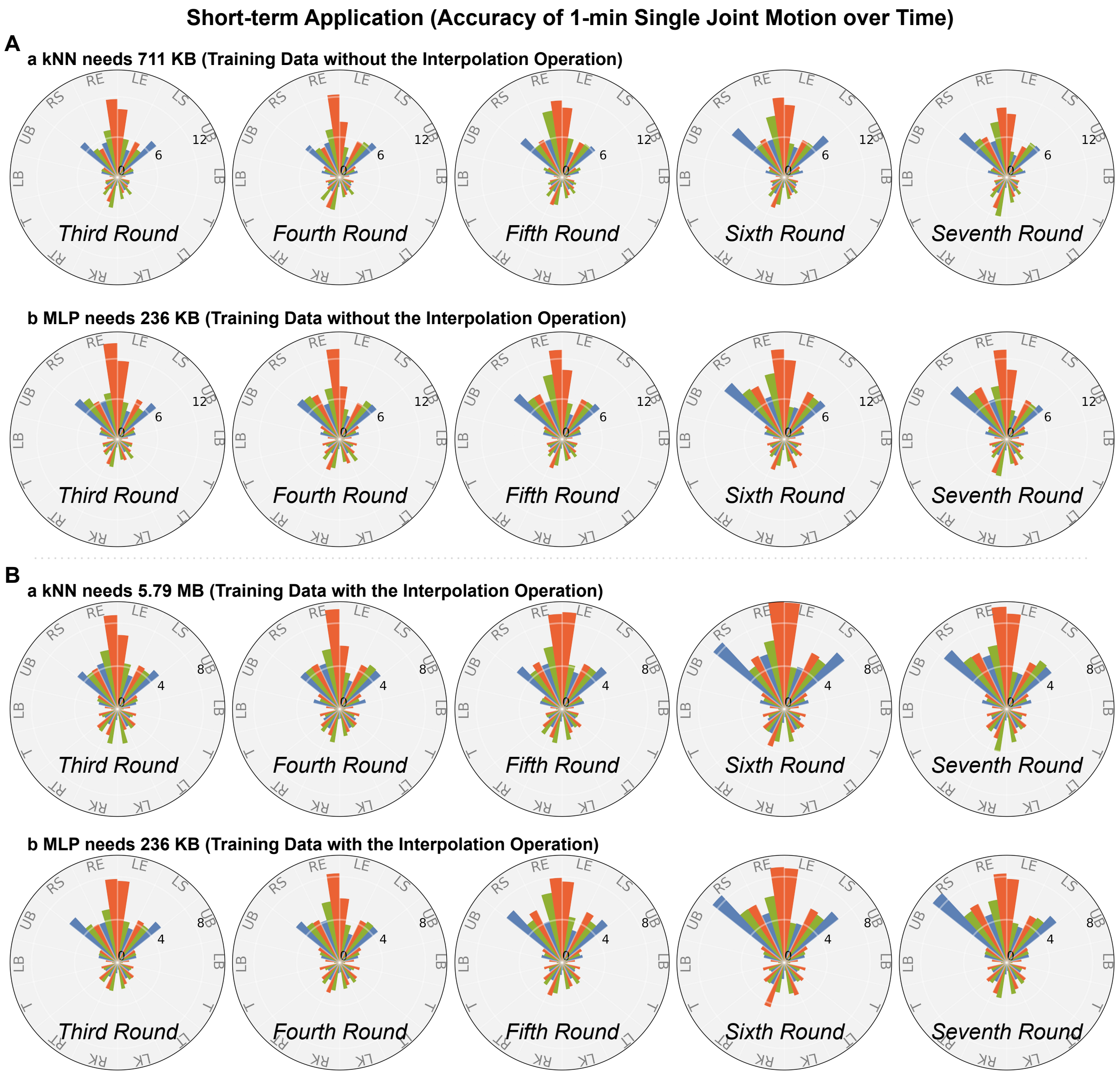

Fig. S 4. Ablation Study for Short-term Application. A, Performance comparison between kNN and MLP on the non-interpolated dataset. **B**, Performance comparison between kNN and MLP on the interpolated dataset.

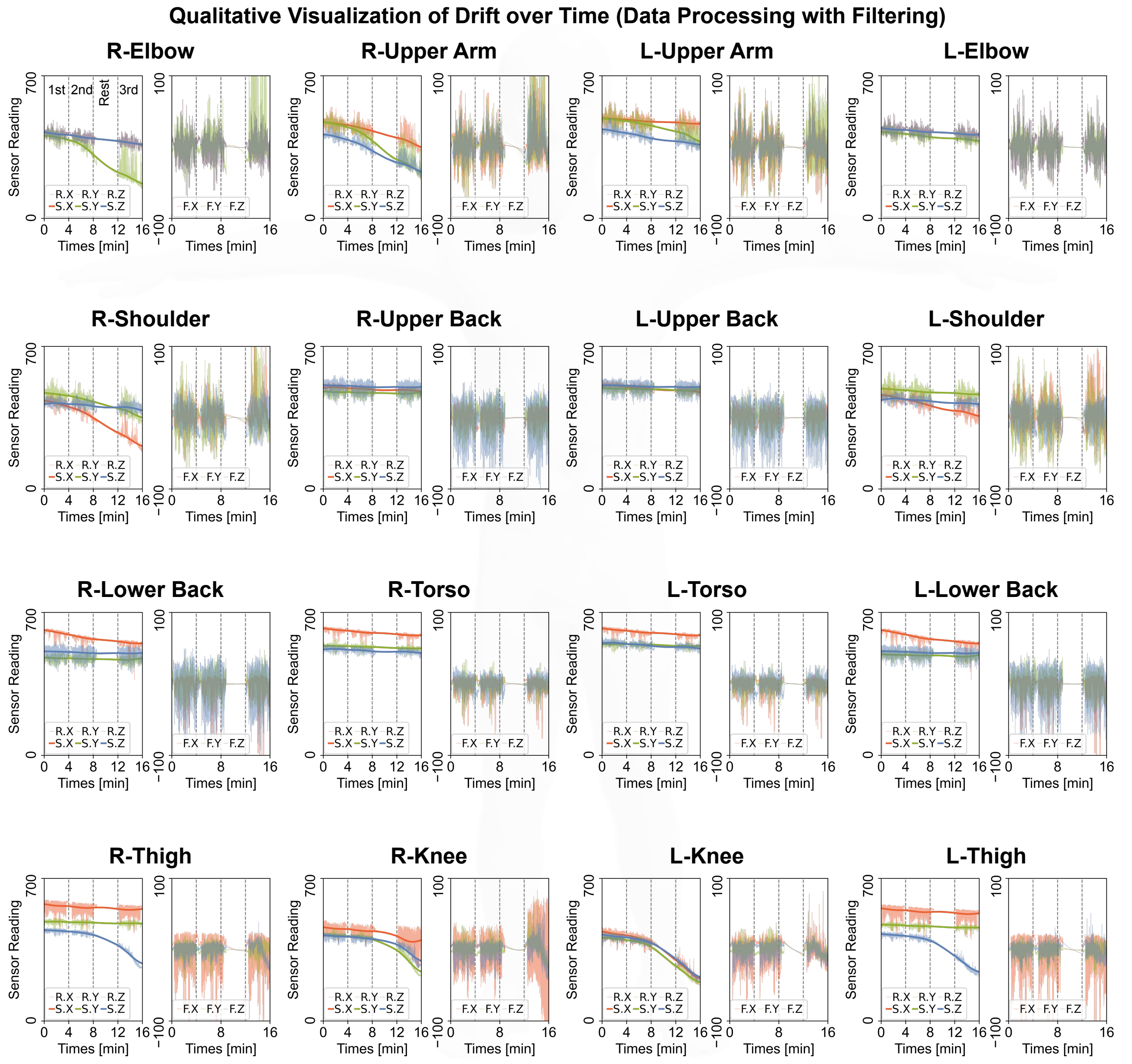

Fig. S 5. Drift Analysis over Time. Each joint is equipped with X (red), Y (green), and Z (blue) sensors to measure the corresponding joint orientations in the X, Y, and Z axes. The solid, thick red, green, and blue lines represent the smoothed curve, highlighting sensor drift. The plot on the right of each joint figure shows a drift-filtered curve, which reflects the sensor reading changes induced purely by motion.

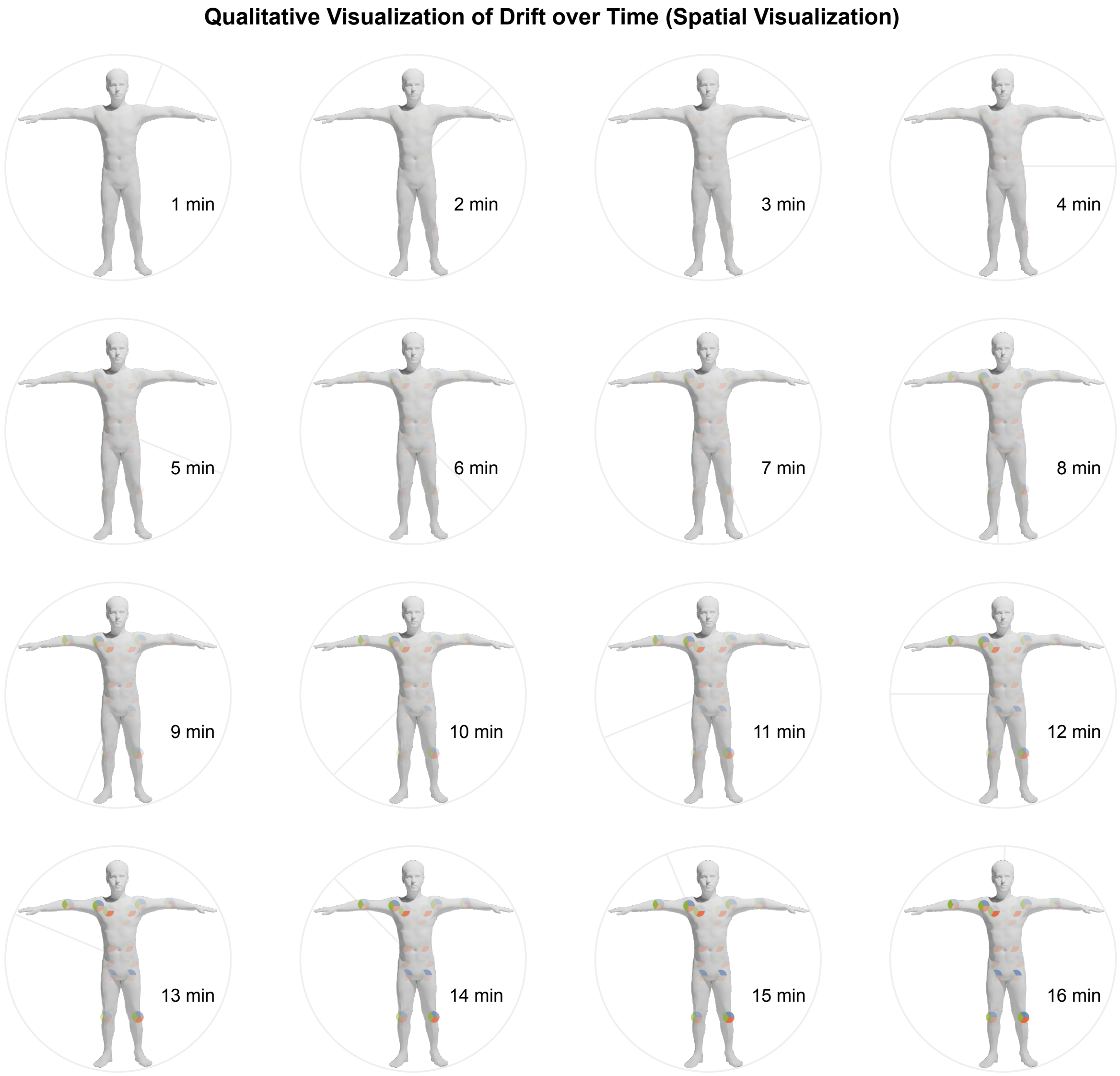

Fig. S 6. Spatial Distribution of Drift over Time. A three-part pie chart, colored in red, green, and blue, represents the drift of X, Y, and Z local sensors over time. Darker colors indicate higher levels of drift.

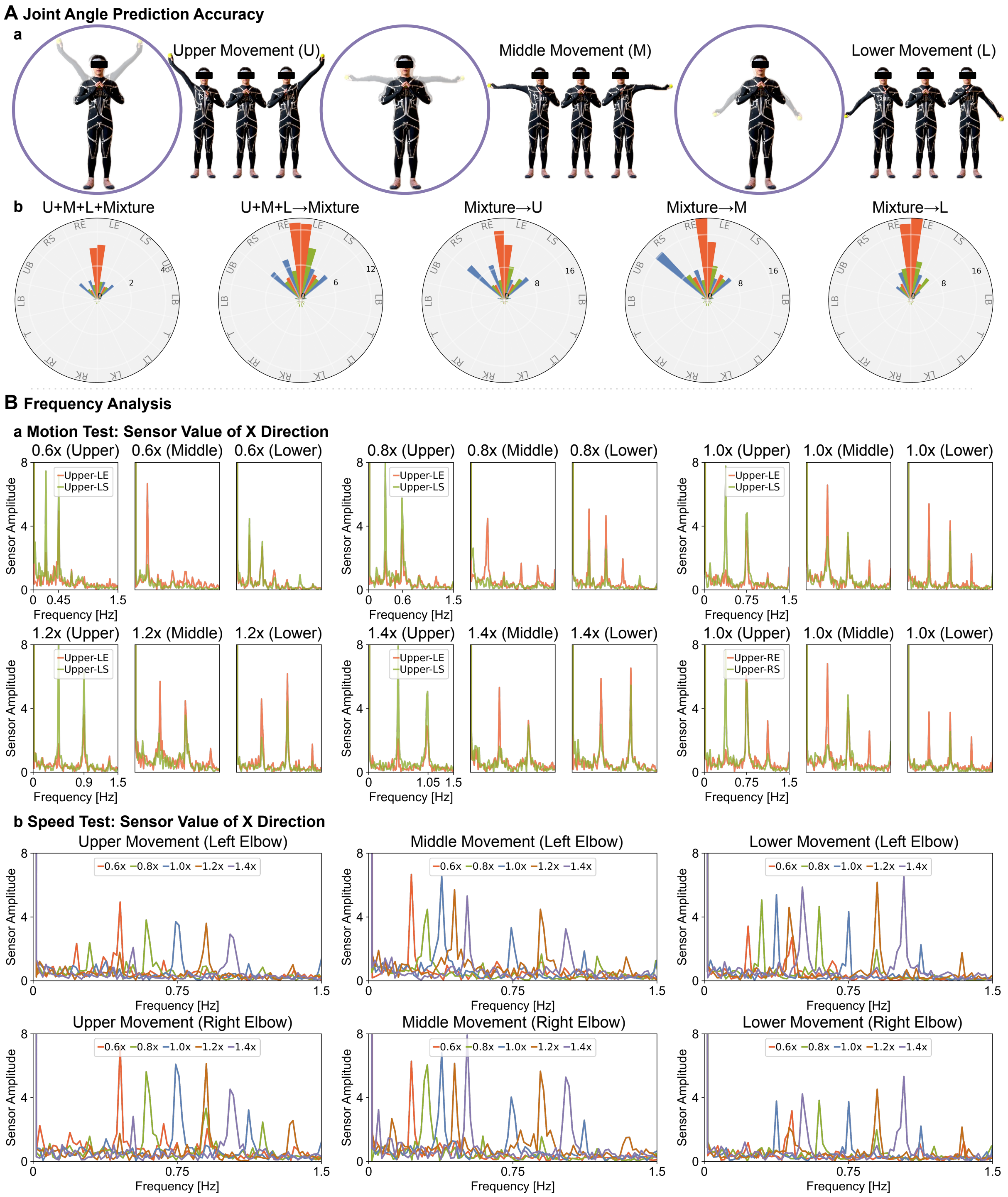

Fig. S 7. Upper Body Pick & Place Accuracy Study and Frequency Analysis. **A**, **b** Ablation study for accuracy assessment at **a** upper, middle, and lower movement heights. **B**, Frequency analysis examining **a** motion height and **b** motion speed.

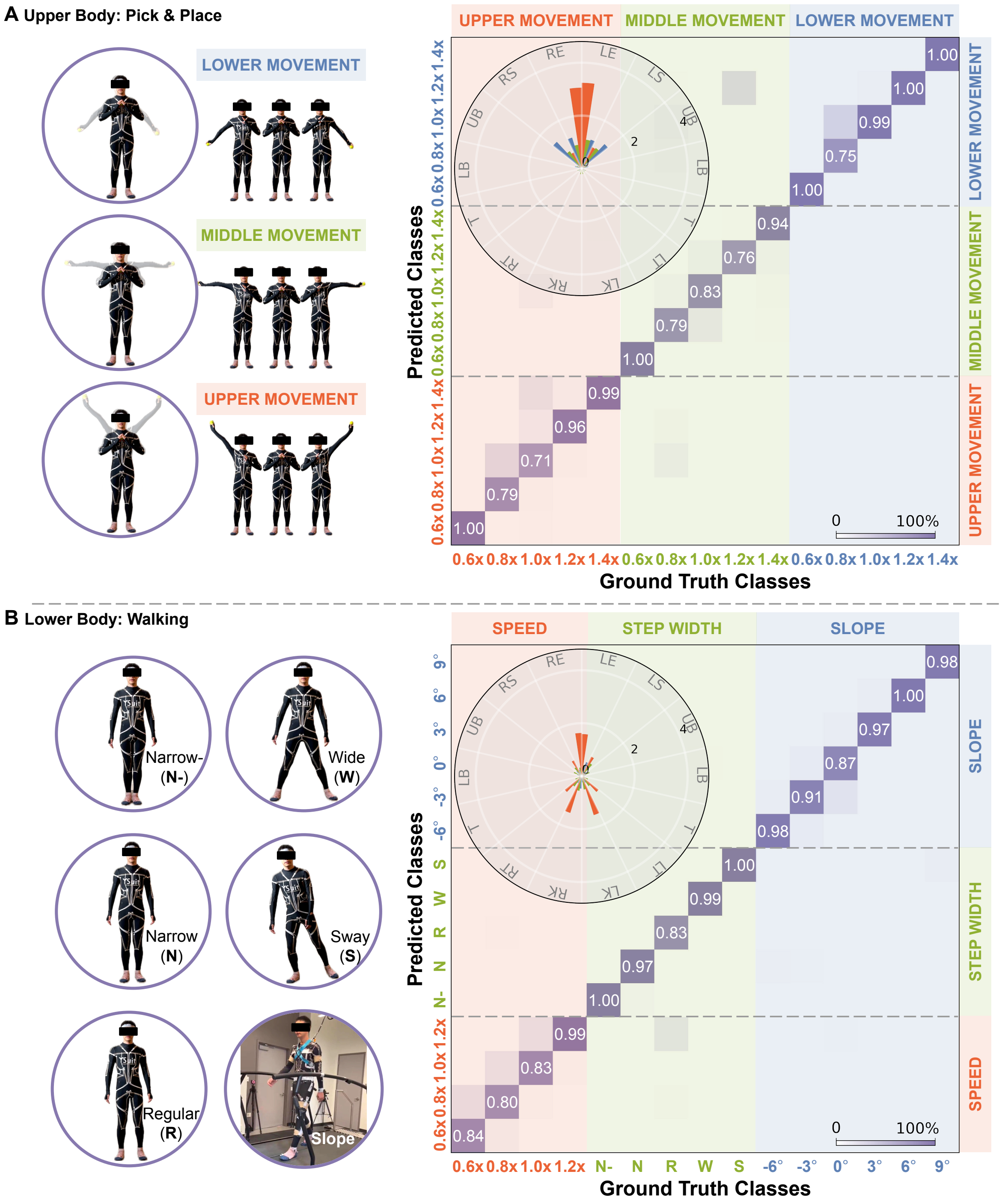

Fig. S 8. Comprehensive Pattern Recognition. **A**, Upper-body pick-and-place tasks are performed at lower, middle, and upper movement levels with speeds of 0.6x, 0.8x, 1.0x, 1.2x, and 1.4x (1.0x = 100% speed at 0.375 Hz). The pose angle accuracy distribution for these tasks at 1.0x speed is shown in the upper-left corner. The pattern classification accuracy (1.00 representing 100% accuracy) of across 15 classes is presented as a confusion matrix. **B**, Lower-body walking tasks include Narrow- (N-), Narrow (N), Regular (R), Wide (w), Sway (S) gaits, with speeds of 0.6x, 0.8x, 1.0x, and 1.2x (1.0x = 100% speed at 1.18 m/s). The pose angle accuracy distribution for lower body walking tasks at 1.0 speed is shown in the upper-left corner. Combining these gaits with six slope levels of -6°, -3°, 0 °, 3 °, 6 °, and 9°, the pattern classification accuracy across 15 classes is evaluated as a confusion matrix.

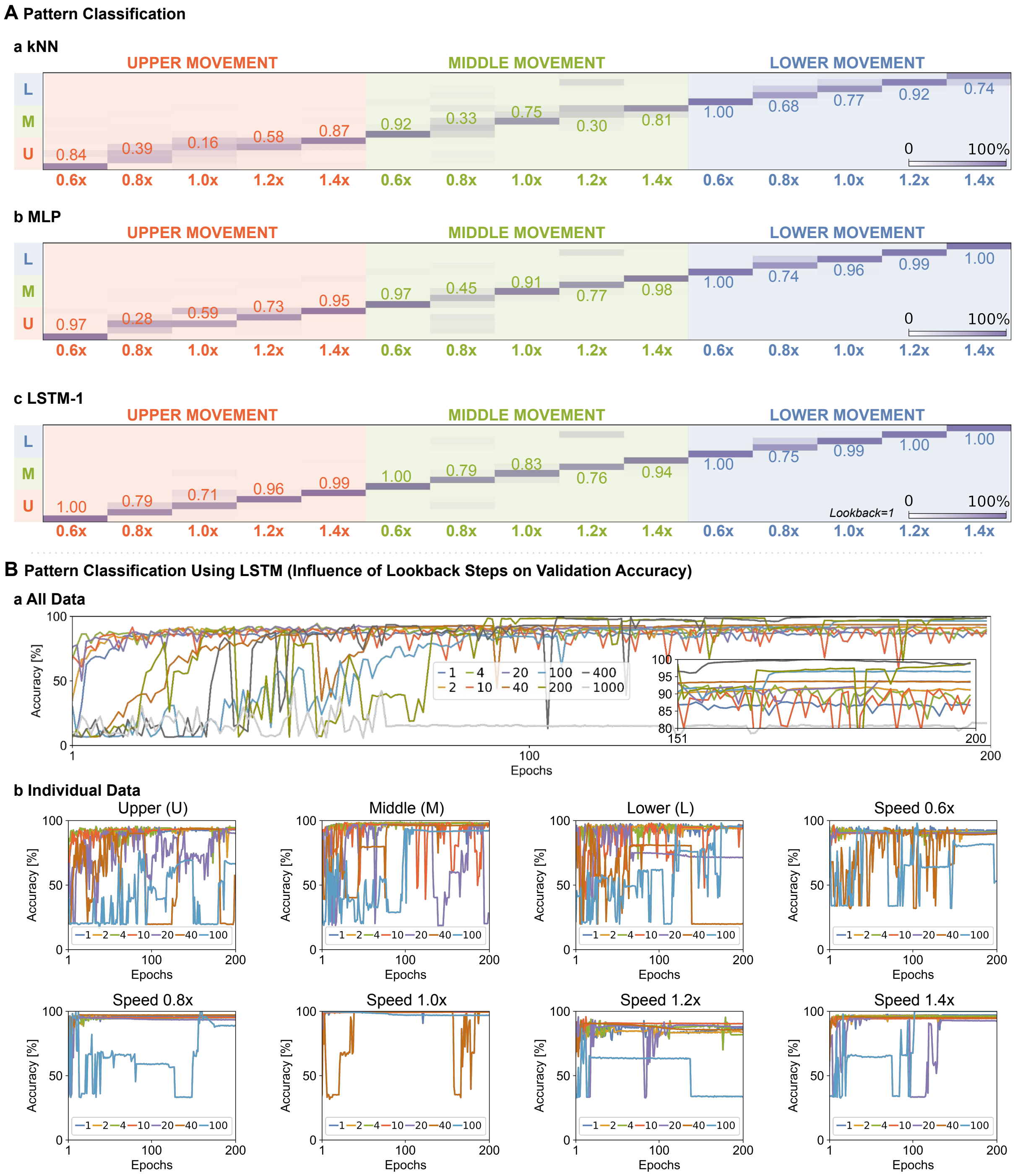

Fig. S 9. Ablation Study for Upper Body Motion Recognition. **A**, Comparison of different pattern classification methods based on classification accuracy: **a** kNN, **b** MLP, and **c** LSTM-1. **B**, Effect of lookback steps on validation accuracy for **a** all motion data samples and **b** individual motion data samples.

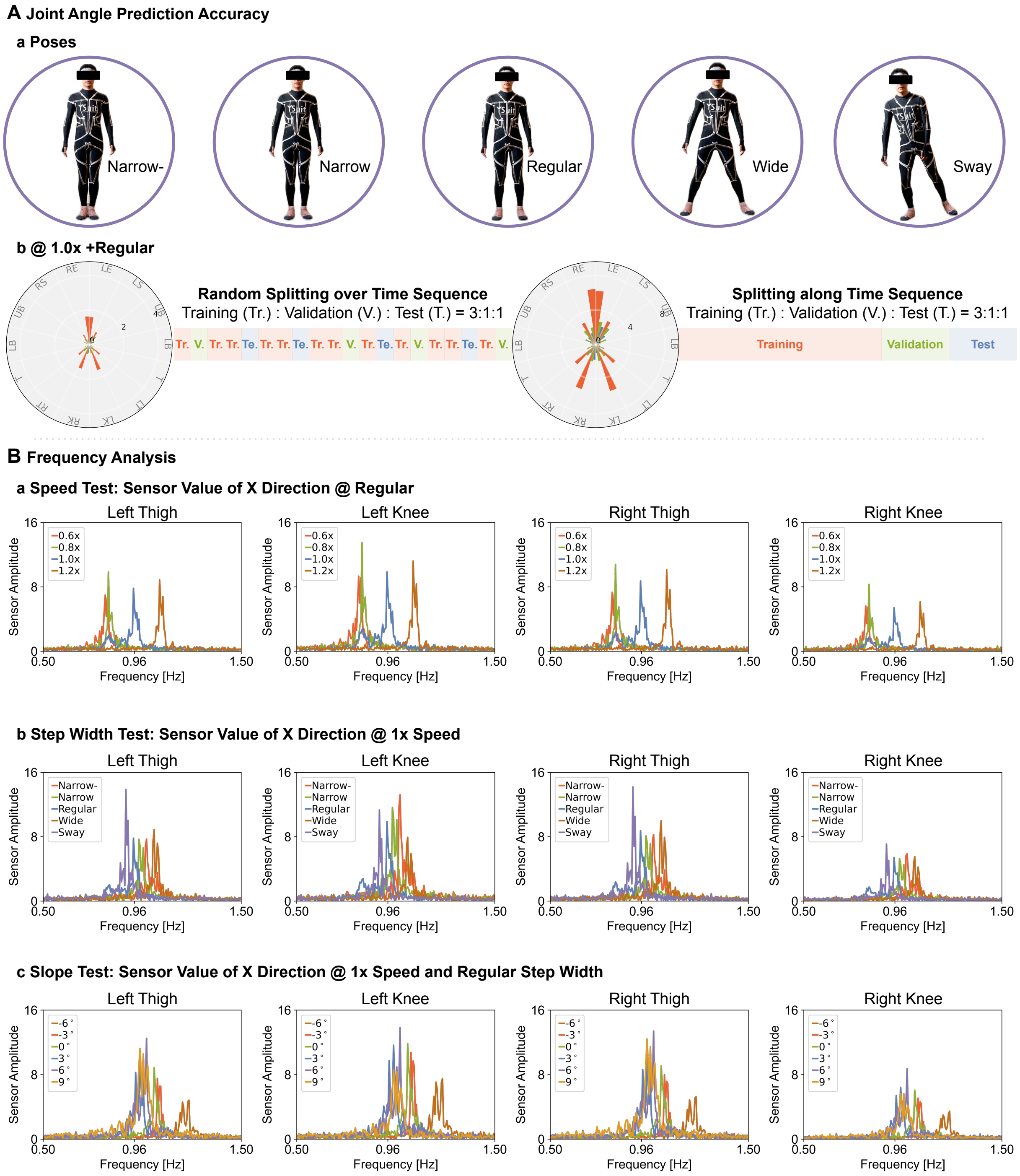

Fig. S 10. Lower Body Walking. A, **a** Different walking step widths and **b** accuracy comparison between two data splitting methods. **B**, Frequency analysis considering **a** walking speed, **b** step width, and **c** slope.

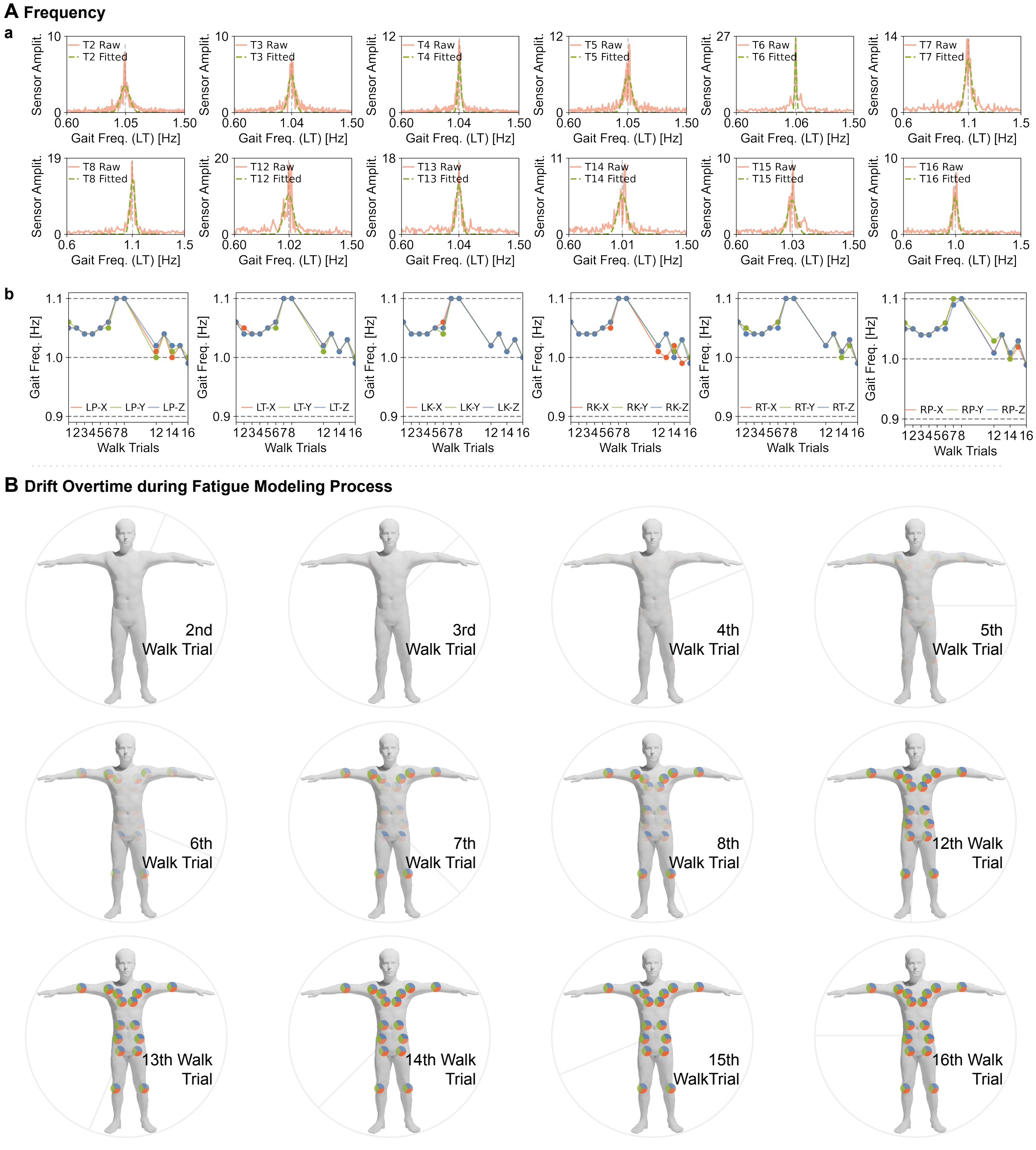

Fig. S 11. Fatigue Modelling. **A**, Frequency analysis conducted during the fatigue procedure. **B**, Spatial distribution of sensor drift over time. A three-part pie chart, colored red, green, and blue, represents the drift of X, Y, and Z local sensors, respectively. Darker colors indicate greater drift.

Movie S1

An introductory video summarizing the key findings presented in the manuscript. This video provides a visual overview of YSuit, covering its concepts, applications, methodologies, and results, making the content accessible to a wider audience.
